# Emergence of recombinant *Mayaro virus* strains from the Amazon basin

**DOI:** 10.1038/s41598-017-07152-5

**Published:** 2017-08-18

**Authors:** Carla Mavian, Brittany D. Rife, James Jarad Dollar, Eleonora Cella, Massimo Ciccozzi, Mattia C. F. Prosperi, John Lednicky, J. Glenn Morris, Ilaria Capua, Marco Salemi

**Affiliations:** 1 Emerging Pathogens Institute, University of Florida, Gainesville, FL USA; 2 Department of Pathology, Immunology and Laboratory Medicine, College of Medicine, University of Florida, Gainesville, FL USA; 3 Department of Infectious, Parasitic and Immune-Mediated Diseases, Istituto Superiore di Sanità, Rome, Italy; 4 Unit of Clinical Pathology and Microbiology, University Campus Bio-Medico of Rome, Rome, Italy; 5 Department of Epidemiology, University of Florida, Gainesville, FL USA; 6 Department of Environmental and Global Health, College of Public Health and Health Professions, University of Florida, Gainesville, FL USA; 7 Department of Medicine, College of Medicine, University of Florida, Gainesville, FL USA; 8 One Health Center of Excellence, University of Florida, Gainesville, FL USA

## Abstract

*Mayaro virus* (MAYV), causative agent of Mayaro Fever, is an arbovirus transmitted by *Haemagogus* mosquitoes. Despite recent attention due to the identification of several cases in South and Central America and the Caribbean, limited information on MAYV evolution and epidemiology exists and represents a barrier to prevention of further spread. We present a thorough spatiotemporal evolutionary study of MAYV full-genome sequences collected over the last sixty years within South America and Haiti, revealing recent recombination events and adaptation to a broad host and vector range, including *Aedes* mosquito species. We employed a Bayesian phylogeography approach to characterize the emergence of recombinants in Brazil and Haiti and report evidence in favor of the putative role of human mobility in facilitating recombination among MAYV strains from geographically distinct regions. Spatiotemporal characteristics of recombination events and the emergence of this previously neglected virus in Haiti, a known hub for pathogen spread to the Americas, warrants close monitoring of MAYV infection in the immediate future.

## Introduction

The *Alphavirus* genus consists of several well-known human pathogenic viruses, the most famous being *Chikungunya virus* (CHIKV)^1^. An emerging member of this genus, *Mayaro virus* (MAYV), is the causative agent of Mayaro Fever, a Dengue and Chikungunya Fever-like infection that manifests as an acute febrile illness often accompanied by severe and prolonged arthralgia^2, 3^. MAYV was first isolated in 1954 in Trinidad and Tobago from five febrile rural workers^4^. Since its first isolation, MAYV has been observed to be endemic and enzootic in Pan-Amazonian countries surrounding central South America, a region characterized by extensive tropical forest^5^. Although previously thought to be exclusive to the Amazon rainforest, Central America^6, 7^, and Trinidad and Tobago^5^, recent cases of MAYV infections have also been reported in Mexico and Haiti^8, 9^, suggesting the virus may be extending its reach.

Previous phylogenetic studies using whole-genome sequencing have classified MAYV strains into two major genotypes, genotype D (widely dispersed) and L (limited), and a third minor genotype, N (new)^5^, consisting of a single sequence isolated from Peru in 2010^5^. Although not observed outside the borders of South America or Trinidad and Tobago^5^, Genotype D currently occupies a vast area from Trinidad and Tobago to Brazil, Peru, Bolivia, and Venezuela^5^, with several available full-genome sequences. Contrastingly, at the time of writing, only a limited number of genotype L isolates have been fully sequenced, spanning an extensive geographical and temporal range that consist of four isolates obtained between 1955–1991 from Pará, Brazil, and two recent isolates from São Paulo state, Brazil, and Haiti^5, 8, 10^. Due to geographical proximity, similar climate, mosquito vector species, and the flux of human population as a result of tourism and immigration^11^, the MAYV case in Haiti poses a potential threat of spread to the southern United States of America (USA), as previously observed for vector-borne viruses such as CHIKV and *Zika virus* (ZIKV)^12–14^. Increased likelihood of spread to other geographical locations is also facilitated by the extensive host plasticity of MAYV, able to infect a wide range of vertebrates, including marsupials and primates, which are the suspected reservoirs for the maintenance of MAYV zoonosis in the rainforest^6, 15, 16^. Within the confines of the rainforest, the main vector for MAYV has been identified as the diurnal, canopy-dwelling *Haemagogus* species (*spp.)* of mosquito - the same vector responsible for transmission of yellow fever virus (YFV) in southern Brazil^16, 17^. The cycle of the vector population and the onset of the Amazon rainy season have previously been associated with MAYV epidemics^18^. However, other species of mosquito have been shown to harbor the virus or exhibit competency as a transmission vector^16, 19, 20^, including *Aedes aegypti* ^21^, the genera responsible for transmission of the recent ZIKV epidemic. Although MAYV titers found in viremic humans are insufficient for efficient transmission by *Ae. aegypti* ^21^, the ability to infect the urban-dwelling *Aedes spp*. provides the opportunity for expansion of the virus into more populated areas and increases the potential threat of a new epidemic^11, 22^.

Similar to that of other alphaviruses, the MAYV genome is a positive, single-stranded RNA molecule of approximately 11–12 kilo bases (kb), encoding two precursor polyproteins, which consist of four nonstructural proteins (nsP1, nsP2, nsP3, nsP4) involved in virus replication and pathogenesis, and five structural proteins (CP, E3, E2, 6k/TF, E1)^23, 24^. Recombination has played an important role as a mechanism of diversification and evolution for RNA viruses, resulting in new species, genera, or even new families, including the chimeric ancestors of the present-day alphavirus clade^1, 25, 26^. Genomic recombination has also been observed previously within the alphavirus genera^27–29^, as phylogenetic analyses have indicated that Sindbis and Eastern equine encephalitis viruses gave rise to the Western equine encephalitis virus complex^25^. More recently, intra-species recombination, potentially involved in the cross-species transmission process, was identified for CHIKV based on nucleotide identify and phylogenetic tree incongruence analyses^27^. However, the extent of intra-species recombination for MAYV has not been investigated, nor the role of recombination in evolution and its relationship with MAYV spread among human populations. Here, we present the first in-depth evolutionary study of MAYV strains isolated in the last sixty years from the Amazon basin^5^, São Paulo State^10^, and Haiti^8^, with a focus on the role of recombination, natural selection, and human mobility in the spread of the virus across central South America and the Caribbean.

## Results

### Recombination of Brazilian MAYV strains resulted in a previously undetected MAYV hybrid L/D genotype

**Figure 1:**
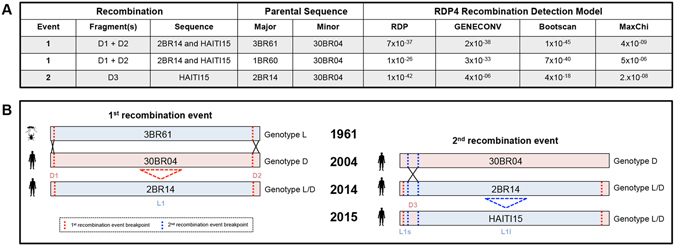
Schematic representation of the identified recombination events associated with MAYV variants 2BR14 and HAITI15. (**A**) Identified recombinant sequences and fragments using various detection models. Significant p-values are indicated (alpha = 0.05), whereas ‘NS’ denotes the absence of statistical support (not significant). First recombination event based on dataset excluding 3BR61 sequence. (**B**) The first recombination event between the minor parental 30BR04 (red) and the major parental 3BR61 (green) gave rise to the recombinant strain 2BR14; the second recombination even between minor parental, 30BR04, and the major parental, the recombinant 2BR14, resulted in the recombinant strain HAITI15. Dotted blue lines indicate genomic breakpoints. The source of isolation of the strains is indicated with the schematic human, mosquito and tick black icons. Schematic representations are based on the results of recombination analysis in RDP4.

The presence of significant conflicting phylogenetic signals in sequence data, due to presence of recombination, homoplasy, model heterogeneity, or sampling error makes impossible to represent evolutionary relationships with a single strictly bifurcating tree. In such cases, relationships among sequences are more accurately described by network-like graphs, such as Neighbor networks (NNet) that can be inferred from genetic distances with a split decomposition-based algorithm^30^. Specific tests, like the pairwise homoplasy index (PHI) test^31^, can also be used to assess whether the conflicting evolutionary relationships in NNet are indeed the result of recombination. NNet and the PHI test detected strong signal for recombination (PHI test p*-*value < 10^−99^) in the MAYV full genome alignment (Figure S1)^32, 33^. Within the NNet, extensive network-like connectivity was observed specifically for genotype L sequences, indicating a relatively high level of conflicting phylogenetic signal within the genotype L clade (Figure S1). Two sequences, referred to as 2BR14 and HAITI15, were identified as putative recombinants, as removal of these sequences resulted in a more tree-like structure of the NNet with bifurcating nodes and a non-significant (0.63) p-value (Figure S1). Recombinant variants 2BR14 and HAITI15 were both obtained from human subjects, the former isolated from a Brazilian patient returning to São Paulo from Pará in 2014^10^, the latter from an eight year-old child in Haiti in 2015^8^. Additional recombination analysis using six separate algorithms^34^ provided corroborative evidence for two independent recombination events (Fig. 1A), as well as specific information about the genomic locations of these events (Fig. 1B and Figure S2). The first recombination event resulted in the recombinant genome of 2BR14, consistent with the phylogenetic network analysis described above. This event was characterized by two recombination breakpoints resulting in the exchange of two relatively small genomic fragments located within the 5′ and 3′ regions, hereafter referred to as “D1” and “D2”, based on genotype composition. The major parental strain involved in this first recombination event, defined as contributing the larger (96.7%) fragment (“L1”) of the final recombinant sequence, was identified as 3BR61, a variant classified as genotype L^5^ isolated from a tick belonging to the *Ixodes spp*. in 1961 Pará, Brazil, and exhibiting 99.2% nucleotide similarity to the recombinant strain isolated in São Paulo more than 50 years later. Moreover, 3BR61 also shared 99.8% similarity with isolate 1BR60, sampled from the *Haemagogus spp*. of mosquito and from the same region (Pará, Brazil) less than one year prior, suggesting similar host adaptation^5^. Removal of the tick-derived sequence (3BR61) during the six independent recombination analyses resulted in the alternative role of its mosquito-derived relative (1BR60) as the major parental sequence to the first recombination event (p-values 4 × 10^−09^–1 × 10^−45^). The minor parental strain, defined as contributing the smaller recombinant sequence fragments (D1 and D2), was identified as 30BR04, a variant classified as genotype D and isolated from a human patient in Acre, Brazil, in 2004^35^. The second recombination event (p-values 7 × 10^−09^–1 × 10^−42^) resulted in the recombinant genome of HAITI15, also consistent with the phylogenetic network analysis described above. This event was characterized by the additional exchange of a 5′ genomic fragment (“D3”) between the now recombinant parent 2BR14 (major parent contributing to 92.8% of the genome) and the minor parent 30BR04, also the minor parent of the previous recombination event. Overall, three genotype D-derived recombinant fragments (D1-D3) were incorporated into HAITI15 through recombination, the third fragment dividing the large L1 fragment into two separate fragments, hereafter referred to as L1s (small) and L1l (large). A more easily comprehensible graphic narrative of the above described recombination events is presented in Fig. 1B.

**Figure 2:**
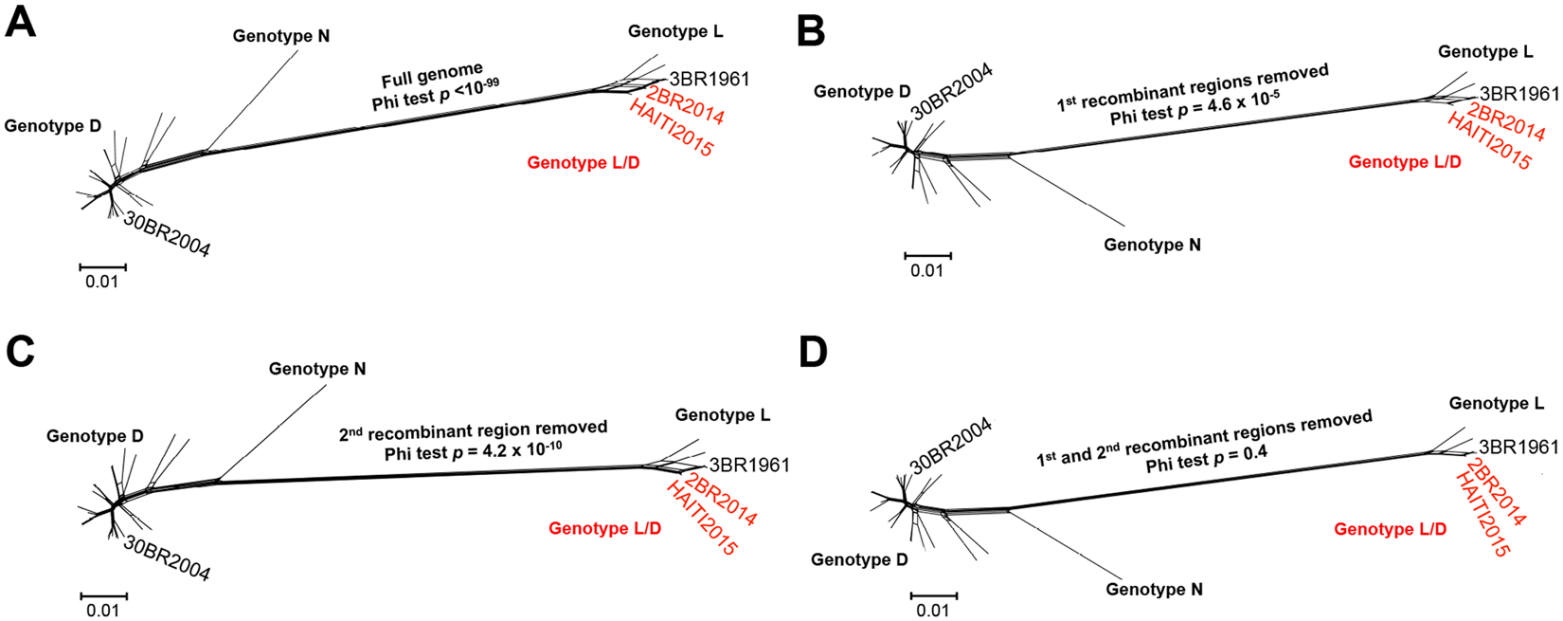
Detection of recombination in the MAYV genome using split-decomposition based networks. Network graphs were generated in SplitsTree based on the (**A**) full genome sequence, (**B**) genome sequence with recombinant regions as a result of the first recombination event removed, (**C**) genome sequence with recombinant region of the second recombination event removed, and (**D**) genome sequence with recombinant regions as a result of the first and second recombination events removed. The *p*-values of the Phi test of recombination for each genomic fragment are reported in each panel.

**Figure 3:**
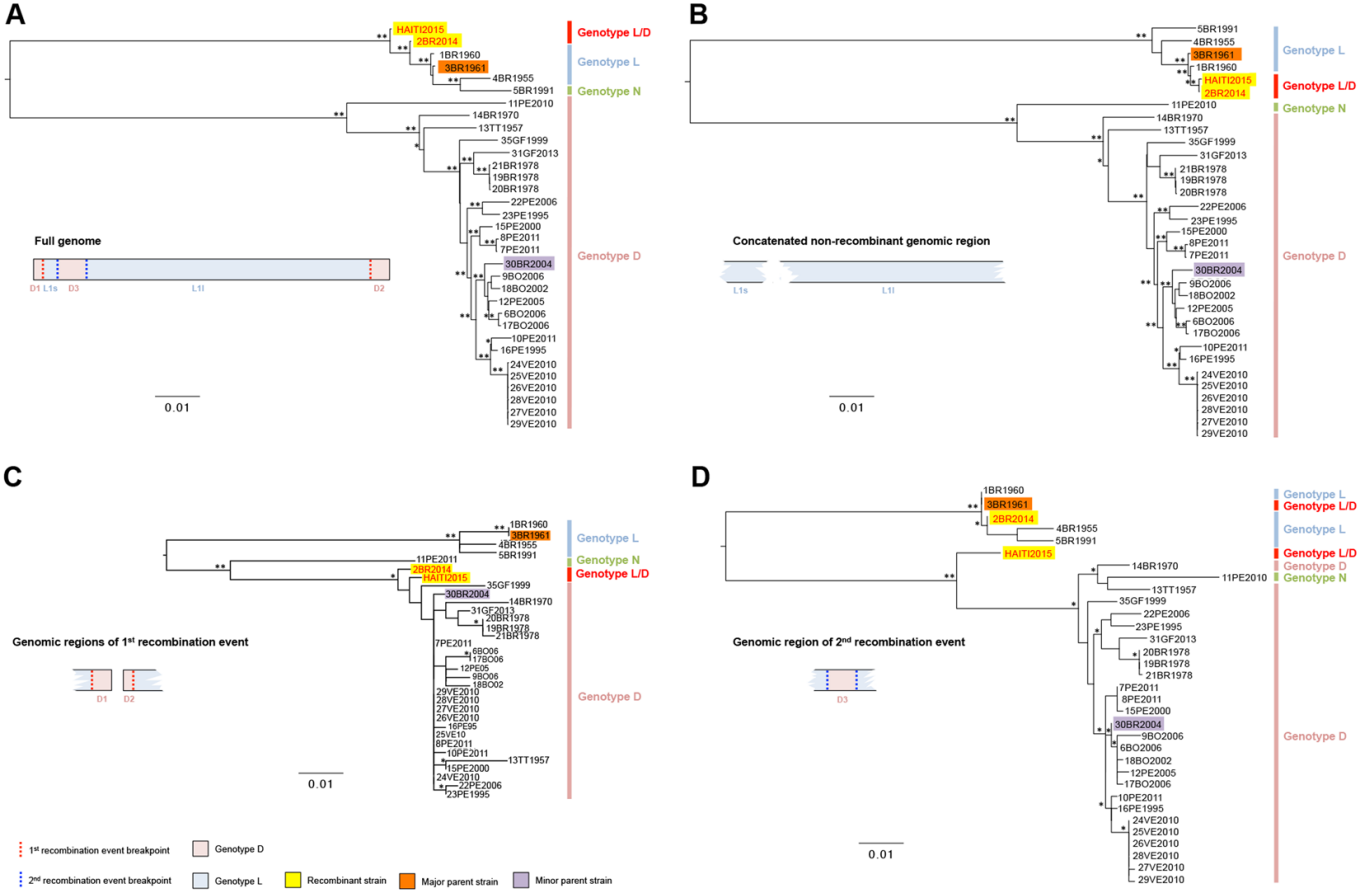
Maximum likelihood (ML) phylogenies of MAYV isolate full-genome, non-recombinant genomic fragment, and recombinant fragment sequences. ML trees were obtained for (**A**) full-genome, (**B**) non-recombinant genomic fragments, (**C**) recombinant fragments resulting from the first recombination event, and (**D**) recombinant fragments resulting from the second recombination event. The recombinant MAYV isolates are indicated by red lettering and highlighted in yellow, whereas the major and minor parental sequences are indicated by black lettering and highlighted in red and orange, respectively. (*) indicates strong statistical support along the branches defined by local or/and standard non-parametric bootstrap (BS) values > 75 or Shimodaira–Hasegawa-like approximate likelihood ratio test (SHa-LRT) > 95; (**) indicates very strong statistical support defined by local or/and standard non-parametric BS > 75 and SHa-LRT > 99.

The occurrence of both recombination events was further confirmed by an additional NNet analysis based on the presence or absence of the identified recombinant regions (Fig. 2A–D). NNet graphs based on full-genome sequences (Fig. 2A), or partial genomes without the recombinant regions resulting from either the first (Fig. 2B) or second (Fig. 2C) recombination events, again presented network-like structure for the genotype L sequences and significant recombination signal (p*-*values < 10^−4^). However, when all recombinant regions were removed (Fig. 2D), the presence of recombination was no longer supported (p*-*value = 0.4). Maximum likelihood (ML) phylogenetic trees inferred from full-genome, as well as sequence fragments corresponding to individual recombination events, also confirmed the presence of recombination (Fig. 3). ML trees inferred from full-genome sequences (Fig. 3A), or from concatenated non-recombinant regions of the MAYV sequences (L1s and L1l) (Fig. 3B), showed strong statistical support for the clustering of recombinant sequences 2BR14 and HAITI15 with genotype L variants, albeit their phylogenetic relationship with the major parental strain (3BR1961) was different in the two trees. The observed clustering pattern was expected as the major parental sequence (3BR61), contributing >90% of the genome, belongs to genotype L. Phylogenetic inference based on the recombinant fragment from the first recombination event (D1 and D2) showed, as expected, 2BR14 and HAITI15 variants clustering with high support within the genotype D clade (Fig. 3C); the genomic fragment from the second recombination event (D3) also confirmed that this fragment, in the HAITI2015 strain, was derived from genotype D (Fig. 3D).

### Evolution of the recombinant genes in South American and Haitian MAYV strains is characterized by episodic diversifying selection

In order to evaluate whether the sampled MAYV recombinant strains exhibited increased fitness, a quantitative codon selection analysis and analysis of co-variation of nucleotide sites in recombinant and non-recombinant genes was performed (see Supplementary Methods). The analysis focused specifically on the two genes involved in the recombination events: *nsP1* and *E1*, which encode enzymes critical in alphavirus RNA synthesis^36, 37^ and viral fusion with the host cell^38^, respectively.

Both genes showed evidence of strong background purifying selection (posterior probability >0.90; Table 1), consistent with previous observations for other arboviruses infecting multiple vertebrate and invertebrate hosts^39–41^. For both genes, negatively selected sites were distributed between both the non-recombinant and recombinant fragments, although with greater prevalence in the non-recombinant regions (Table 1). Alternatively, episodic diversifying selection (EDS) was detected in *nsP1* but not *E1* (Table 1). By using a method robust to the presence of recombination^42^ (see Supplementary Methods), the *nsP1* L1l fragment was identified as the one with the greatest number of sites (seven) evolving under EDS. As transient bursts, or sweeps, in selection indicate adaptive change^43^, the result suggests a role for *nsP1*, specifically the 3′ region, in adaptation. In order to determine whether EDS was associated with recombination, we used a branch-specific model with no *a priori* specifications^44^ (i.e., each branch had an equal probability of exhibiting this pattern of selection, see Supplementary Methods). Although branch-specific EDS was not detected along the branches leading directly to the recombinant viral variants (nor any other individual branches), when amino acid changes at EDS sites were mapped along the branches of the ML genealogy, the branches leading to the two major parental sequences both contained sites belonging to this classification (Figure S3), suggesting a potential role for these sites and corresponding selective pressure in facilitating recombination between the parental strains.

**Table 1:** Evidence of selection pressure among codon sites within the recombinant *nsP1* and *E1* genes.

Method	*nsP1* D1 fragment	*nsP1* D3 fragment	*nsP1* 1l fragment	*E1* L1l fragment	*E1* D2 fragment
FUBAR^a^	0 sites	54 sites with evidence of purifying selection	102 sites with evidence of purifying selection	156 sites with evidence of purifying selection	15 sites with evidence of purifying selection
BUSTED^b^	no evidence of episodic diversifying selection	no evidence of episodic diversifying selection	evidence of episodic diversifying selection	no evidence of episodic diversifying selection	no evidence of episodic diversifying selection
aBSREL^c^	0 out of 9 branches	0 out of 51 branches	0 out of 59 branches	0 out of 43 branches	0 out of 41 branches
MEME^d^	0 sites	2 sites with evidence of episodic diversifying selection: 39, 242	6 sites with evidence of episodic diversifying selection: 355, 356, 384, 386, 388, 466.	2 sites with evidence of episodic diversifying selection: 141, 285	0 sites

^a^Fast unconstrained Bayesian approximation of pervasive selection (PP > 0.90); ^b^Bayesian unconstrained test for episodic diversifying selection (LRT p ≤ 0.05); ^**c**^Branch-specific episodic diversifying (adaptive) selection (LRT p ≤ 0.05); ^d^Site-specific episodic diversifying selection (LRT ≤ 0.05).

An additional measure of selective pressure can be observed via analysis of co-varying, or co-evolving, sites^45^. The introduction of a recombinant region into a protein under strong purifying selection would result in high levels of co-variation, as changes from the introduction of a recombinant fragment typically require a compensatory change in an interacting residue in the non-recombinant region. The majority of co-evolution events were observed within the branches of the *nsP1* genealogy corresponding with genotypes D and N (Figure S4). However, a network of conditionally dependent non-synonymous changes among three codon positions (P144Q, A216, R232K) occurred along the branch leading to the most recent common ancestor (MRCA) of the L/D sequence 2BR14 and the genotype L clade (“Recombinant Branch 2”, Figure S4). The co-varying network of the three *nsP1* sites along Recombinant Branch 2 occurred following the second recombination event (incorporation of adjacent genotype D fragments), suggesting some form of adaptation to the modified genome. In other words, analysis of long-range co-variation might indicate an interaction between this region of the protein (acquired from genotype D following the second recombination event) and another region outside of *nsP1* maintained from genotype L. Although none of these three sites coincided with the sites detected as experiencing EDS, this does not exclude a co-dependence at the protein level and its implication in the process of recombination-driven diversification.

### MAYV strains exhibit optimal adaptation to the human and *Aedes aegypti* genetic code

**Figure 4:**
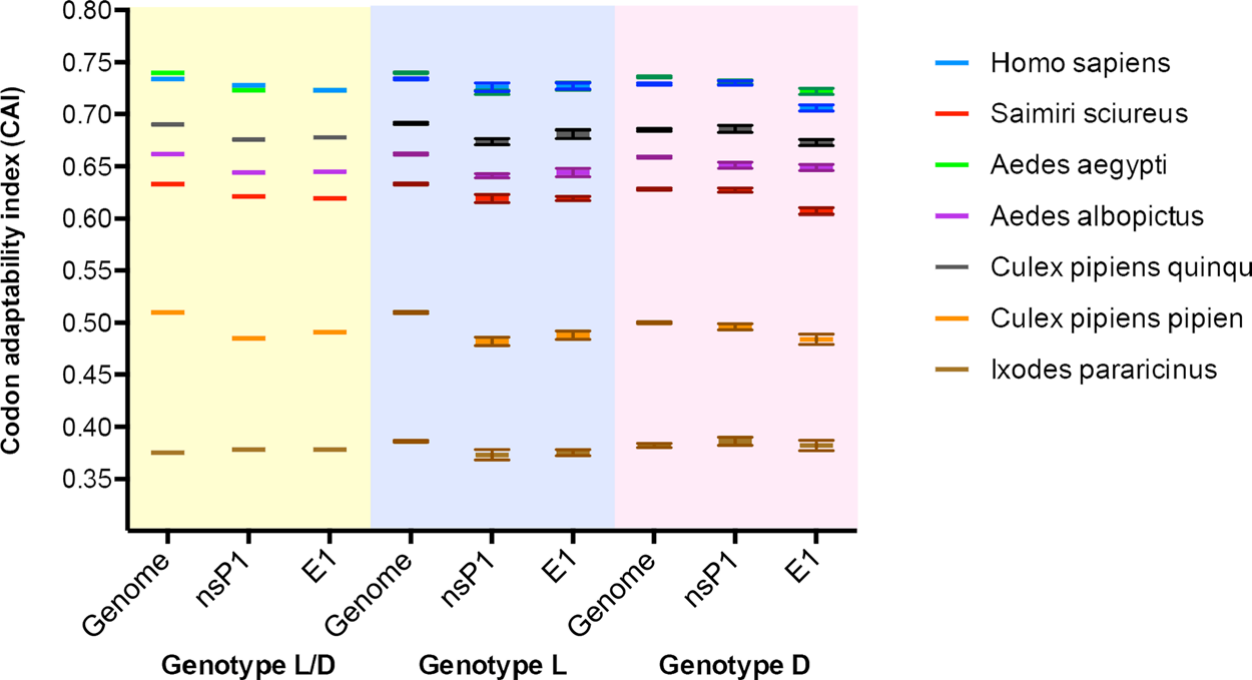
Codon adaptability index (CAI) analysis for MAYV full-genome and gene sequences based on the codon usage of the most frequent genes of various host and vector species. CAI values based on full-genome, and nsP1 and E1 genes, reflecting the adaptation of MAYV genotypes L/D (yellow shade), L (blu shade), and D (pink shade) to human and non-primate hosts, and arthropod vectors. Values are reported as mean ± standard deviation, with the exception genotype L/D, for which only two sequences are available.

In order to investigate the impact of recombination on MAYV adaptation to pre-specified mammalian hosts and arthropod vectors, we compared codon adaptation index (CAI) estimates based on the codon usage bias of MAYV genes and full-genomes (Fig. 4). First, we compared the codon usage of the recombinant MAYV genotype with non-recombinant genotypes against the codon usage of *Homo sapiens*, as well as the non-human primate species, *Saimiri sciureus*, native to the tropical areas of South America, as a potential reservoir in the Amazon forest. CAI values for both *H. sapiens* and *S. sciureus* indicated preference of MAYV for the human host. Although more information is needed to determine MAYV adaptability in non-human primate species, CAI values for recombinant and non-recombinant genotypes were similar. A notable exception to this similarity, however, was observed for the genotype D *E1* gene, which exhibited lower values as compared to hybrid L/D and genotype L *E1* genes. This difference suggests that recombination may have enhanced *E1* gene adaptability to humans as compared to genotype D. This finding is of particular importance, given that mutations in *E1* gene have been previously associated with increased transmission and replication fitness^46^.

Next, we compared MAYV adaptation to potential mosquito vectors. Highest CAI values across all mosquito vectors were observed for *Ae. aegypti*, and *Culex quinquefasciatus*, primarily found in urban and more tropical regions, respectively. CAI values for recombinant sequences were very similar to values reported for the non-recombinant sequences, suggesting that recombination did not impact adaptation to these mosquito species.

Considering the unusual and unique isolation of the 3BR61 genome from a tick belonging to the *Ixodes* spp. (personal communication of Dr. Robert B. Tesh and Dr. Scott Weaver), and the potential contribution of the tick-derived sequence to the first recombination event, we tested the adaptability of MAYV to the *Ixodes spp*. of tick, specifically *I. pararicinus*. As expected, CAI values did not differ among recombinant and non-recombinant genotypes, and CAI values for this vector were the lowest across all vectors and hosts tested, indicating poor adaptability of MAYV to *I. pararicinus*. Although we cannot interpret our results in the context of all tick species, our finding is in agreement with previous literature reporting that tick-mediated transmission of alphaviruses, especially when acquired *via* recent blood meal, is less prevalent than other arboviruses, such as flaviviruses^47^. Moreover, these results support the previously stated hypothesis that the highly genetically similar mosquito-derived isolate 1BR60 was the true major parental strain in the first recombination event.

### Temporal emergence of MAYV recombination events

An evolutionary molecular clock was calibrated with the Bayesian coalescent framework to determine the origins of the individual recombination events. Since node age estimates can be affected by recombination^48^, in order to track the emergence of the 2BR14 and HAITI15 strains, we estimated the time of the most recent common ancestor (TMRCA) of the non-recombinant regions of the genome alone, for which strong temporal signal was detected (Figure S5). The mean Bayesian evolutionary rate estimate for the non-recombinant regions, inferred using a strict molecular clock and Bayesian skygrid demographic prior (Table S3), was 6.11 × 10^−5^ substitutions/site/year (ssy) with a 95% highest posterior density (HPD) interval of 3.46 × 10^−5^–9.14 × 10^−5^ ssy. While this estimate is lower than that reported previously (1.67 × 10^−4^ [1.02 × 10^−4^–2.41 × 10^−4^ ssy])^5^, the estimate reported herein was based on a larger data set and evolutionary rate information pooled across individual genomic partitions (genes and non-coding regions), rather than assuming identical evolutionary models for each partition. This method, employed using the Bayesian Skygrid model, has been shown to improve reliability of Bayesian inferences^49^. The TMRCA of all MAYV strains was approximately 145 CE (95% HPD: 699 BCE–773 CE). The TMRCA for the genotype L clade was estimated at 1433 CE (95% HPD: 1183–1633), 1820 CE (95% HPD: 1747–1874) for genotype D. The estimated TMRCAs for the recombinant 2BR14 and HAITI15 strains were, on the other hand, much more recent, both approximately 2007 CE (95% HPD: 2002–2012). Notably, the TMRCAs of major and minor parental strain traced back to the mid-1940s (Table S4), suggesting that those strains may have been circulating at a sub-epidemic level for several decades before the recent recombination events.

### The potential role of human mobility dynamics in facilitating recent MAYV recombination

**Figure 5:**
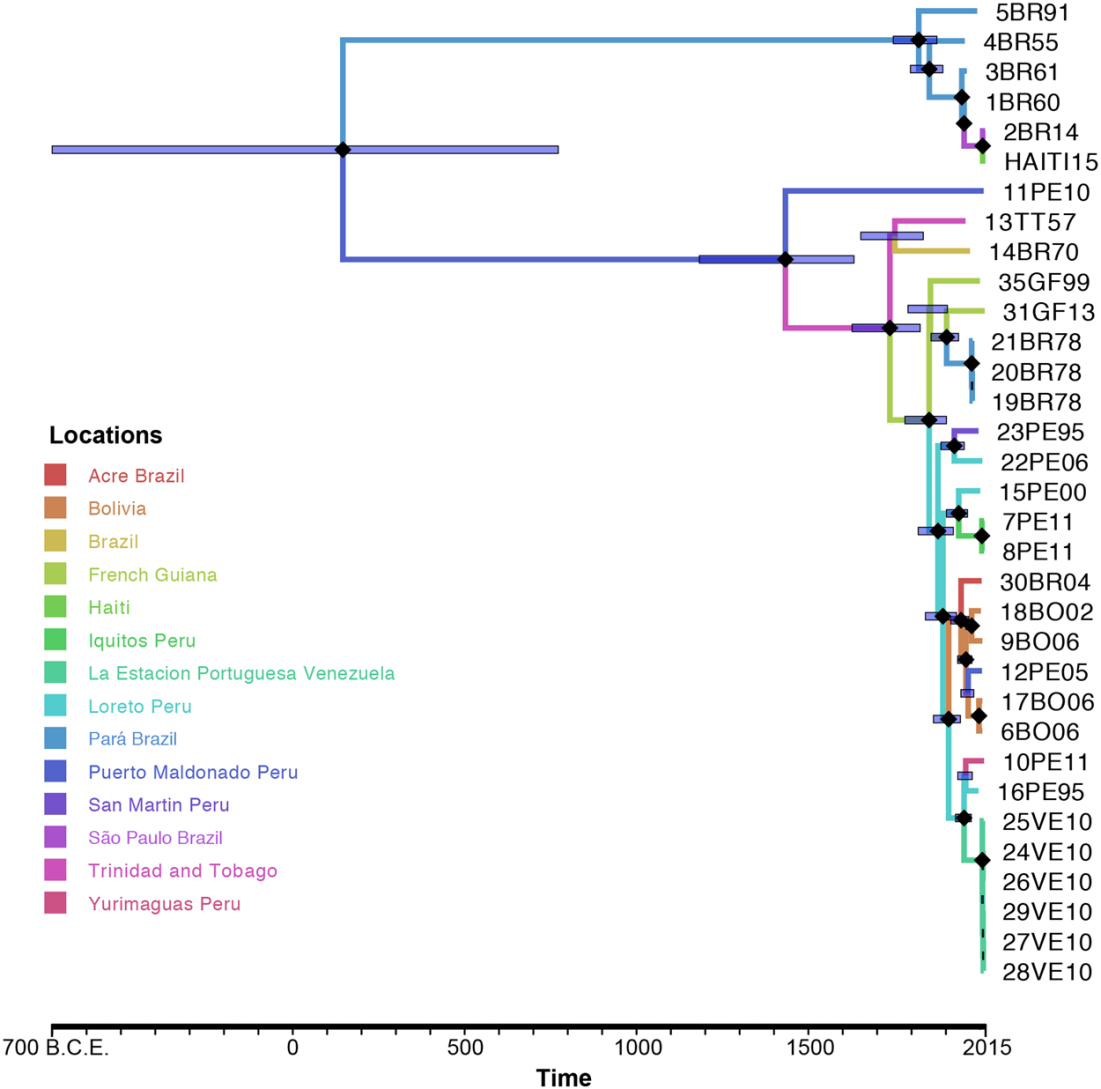
Bayesian phylogeographic analysis of MAYV non-recombinant fragment sequences. Time-scaled phylogenetic maximum clade credibility tree inferred using the Bayesian Skygrid demographic, strict molecular clock, and asymmetric phylogeographic diffusion models, implemented in BEAST v1.8.3. Branches are colored according to geographical location, and black diamonds represent branches supported by posterior probability > 0.99.

The spatial dissemination of MAYV between South American and the Caribbean during 1955–2015 was analyzed within the Bayesian phylogeographic framework^50^ using an asymmetric spatial diffusion model for the partitioned non-recombinant region of the genome (Fig. 5). Despite non-uniform sampling of available sequences from geographical locations, including over-representation of Pará, Brazil and La Estación Portuguesa, Venezuela, linear regression analysis of the relationship between migration rate and sample size (number of sequences) indicated no significant bias of the sampling scheme on the phylogeographic inferences (Figure S6). Results pointed to Puerto Maldonado, Peru, as the ancestral location from where MAYV eventually spread to South America (Table S5). Spatiotemporal reconstruction indicated, however, that MAYV spread from Puerto Maldonado to Trinidad and Tobago and Pará between 1755 and 1847, much later than the estimated TMRCA (145 CE). In agreement with previous inferences of MAYV epidemiology^4, 5, 19, 20, 35^, our results showed MAYV variants belonging to genotype D circulating broadly among South American countries. Between 1900 and 1962, intense interchange of MAYV strains was observed within northern Peru, as well as from southern Peru to Bolivia or Peru to Acre. Moreover, in agreement with previous Bayesian phylogeography studies^5^, our analysis tracked Loreto, Peru, as the origin of the MAYV 2010 outbreak in French Guiana.

**Figure 6:**
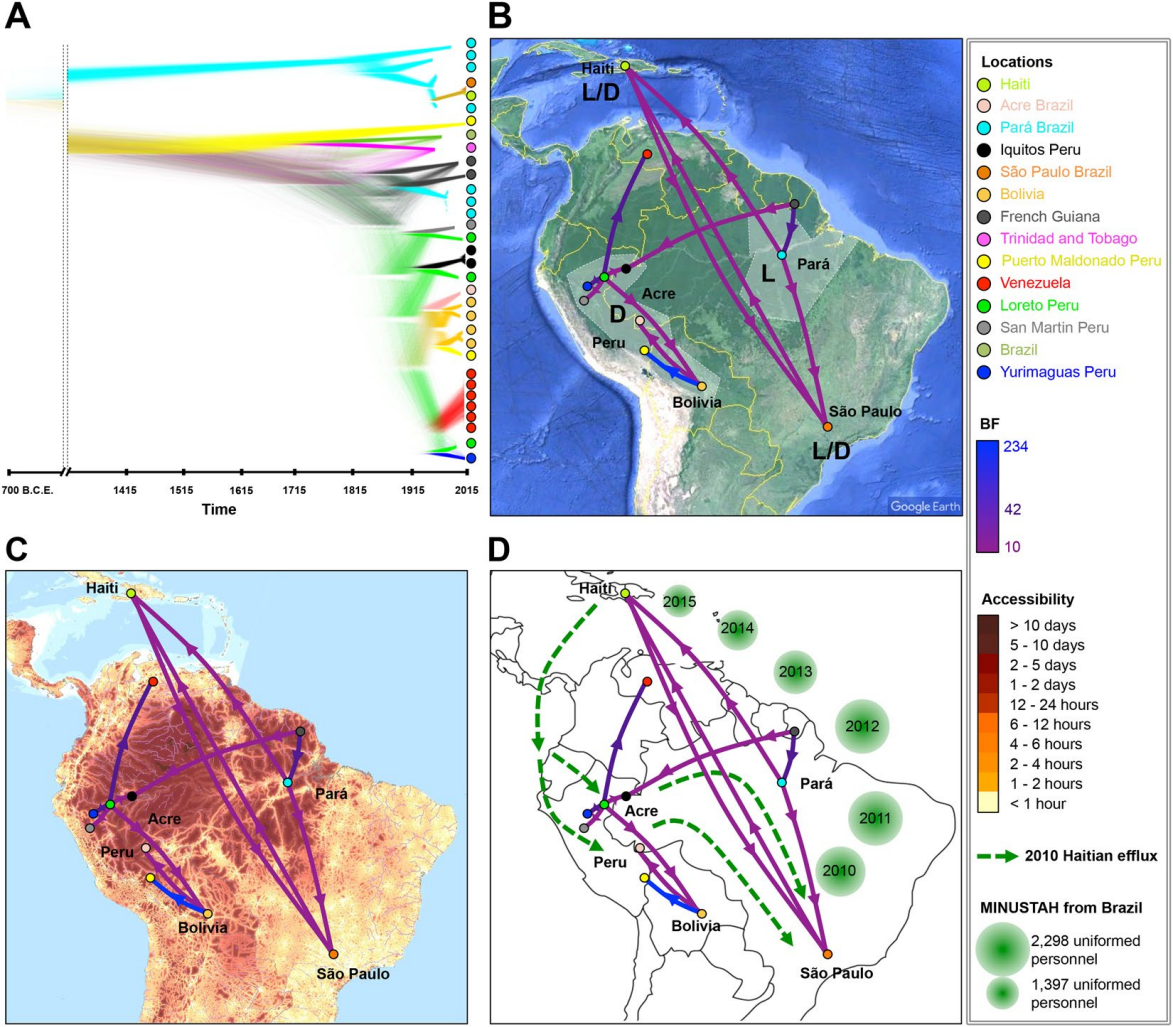
Schematic representation of the relationship between MAYV spread and human population connectivity and migration patterns among central South American and Haiti following the 2010 Haiti earthquake. (**A**) Time-scaled phylogenetic DensiTree representation of the Markov chain Monte Carlo sampled trees from the Bayesian posterior distribution. Branches are colored according to geographical location (legend at right). Well-supported branches are indicated by regions of high density (more solid color), whereas low-density regions (webs) indicate little agreement amongst the trees. (**B**) MAYV migration patterns inferred from Bayesian phylogeographic analysis using a discrete trait asymmetric diffusion model represented using SPREAD. Letters in bold indicate the major genotype circulating in each shaded area. (**C**) MAYV migration patterns superimposed onto the accessibility map of the Amazonian basin, and (**D**) compared to human migration started in 2010 from Haiti to Brazil and Peru, indicated by dotted green lines with directionality. Brazilian contribution to MINUSTAH personnel in Haiti during 2010–2015 is represented by green bubbles, with the size of each individual bubble corresponding to the number of uniformed personnel present during each year. MAYV migration patterns represented in (**B**,**C** and **D**) represent significant non-zero migration rates (Bayes Factor [BF] > 10), and are colored according to BF (legend at right). Maps were obtained from Google Maps (https://mapstyle.withgoogle.com), SPREAD software (www.kuleuven.be/aidslab/phylogeography/SPREAD.html), and ArcGIS database (https://www.arcgis.com) based on a previously published and available dataset (https://tiles.arcgis.com/tiles/P8Cok4qAP1sTVE59/arcgis/rest/services/Accessibility_Travel_time_to_Major_Cities/MapServer).

Spatial diffusion patterns summarized across the Bayesian tree posterior distribution (Fig. 6A) indicated that MAYV initially transitioned from Pará to São Paulo (Fig. 6B), consistent with the observation that the 2BR14 recombinant strain, isolated in 2014, was infecting a Brazilian worker returning to São Paulo from the state of Pará^10^ (Fig. 6B). The virus was subsequently introduced to Haiti from São Paulo, where the second recombinant sequence, HAITI15, was sampled^8^ (Fig. 6B). Since previous studies have shown that spatial accessibility is associated with viral spread^51, 52^, we sought to determine whether accessibility potentially played a role in the spread of MAYV across the Amazonian basin by qualitatively assessing the relationship between the inferred MAYV phylogeographic migration patterns and publicly available human accessibility data (see Supplementary Methods). Accessibility was defined as the travel time *via* land (road or off-road) or water (navigable river, lake, or ocean), which was estimated using a cost-distance algorithm^52^. MAYV genotypes L and D strains have been detected, so far, in distinct regions of the Amazon basin (Fig. 6B), characterized by low spatial accessibility (Fig. 6C). For example, the estimated travel time from the western (Acre) region of Brazil, where genotype L strains circulate, to the eastern (Pará) region, where mainly genotype D strains are found, is greater than 10 days (Fig. 6C). Extensive vegetation of the Amazonian forest and absence of transportation infrastructures may account for low accessibility, which would limit MAYV circulation between specific endemic areas, such Acre and Pará.

Given the unlikely facilitation of recombination by daily human trafficking activity between remote Brazilian regions, we next investigated whether a specific human migration event could have contributed to the inferred diffusion among the sampled locations. Following the earthquake in Haiti in 2010, a large number of refugees of this devastating natural disaster fled to neighboring South America^53^. The immigrants relocated to Brazil following an Ecuadorian route that split off into two separate migration paths to São Paulo - one going through Peru, the other one through Acre and Pará (Fig. 6D). Inferred directional spread of MAYV among sampled locations coincided with the Haitian immigration flux following the 2010 earthquake to Peru and Brazil. United Nations stabilization missions in Haiti, (MINUSTAH) with contributions from more than 100 countries worldwide (including Brazil)^54^, have been ongoing since 2004. In 2011 and 2012, however, the number of uniformed Brazilian personnel occupying Haiti almost doubled relative to 2010 (Fig. 6D)^55^, accommodating the increased necessity for care of earthquake victims and containment of the spread of cholera (http://www.un.org/en/peacekeeping/missions/minustah/emergency.shtml). As of December 2016, MINUSTAH personnel are still present in Haiti, although fewer in number (http://www.un.org/en/peacekeeping/missions/minustah/facts.shtml). The sampling of the Haitian strain in 2015 and observation of MAYV gene flow from apparent end-point refugee destinations (Pará and São Paulo states) to Haiti, may be explained by MINUSTAH occupation of the island (Fig. 6D), possible return of refugees, or a combination of both.

## Discussion

Using in-depth phylogenetic analysis and spatiotemporal reconstruction of the evolutionary history of MAYV sequences collected over the past sixty years, we have provided evidence to support the recent occurrence of separate, but not necessarily independent, recombination events between geographically distinct MAYV strains, potentially resulting in increased human adaptation. The geographic distance and low connectivity between western (endemic to MAYV genotype D) and eastern (endemic to MAYV genotype L) Brazilian states^5^ responsible for the recombinant genotype D/L strain described herein renders recombination an unlikely event, explaining the paucity of MAYV recombination studies. However, based on historical information, we propose that recent changes in human mobility patterns connecting these Brazilian regions may have facilitated recombination between the two MAYV genotypes, as well as their introduction to non-endemic areas such as São Paulo and Haiti^53^. The chance introduction of pathogens in the Caribbean region due to human mobility is not unprecedented^55, 56^, but additional data, specifically increased sampling from the Caribbean, will be required in the future to support fully this hypothesis.

Although the distribution of MAYV migration rate estimates among locations was not significantly biased by a non-uniform geographical sampling strategy, we are aware that incomplete sampling due to the limited number of MAYV full-genome sequences available limits our ability to interpret the potential contribution of missed sampling locations to MAYV spread as well as the temporal estimates of divergence events. Therefore, caution in assuming the identification of the precise location and timing of recombination, without consideration of the dataset as a sample of unknown proportion from the population, is recommended, as with any epidemiological inference. However, given knowledge of restriction of genotype prevalence to the western and eastern regions of the Amazon basin, irrespective of the specific state and/or country, we are confident that the recombination events identified are concordant with the human migratory paths described. The incorporation of additional full-genome sequences, once available, would likely provide a smaller confidence interval of the timing of the recombination events, confirming the relationship of these events with the 2010 Haitian earthquake. Similarly, given an incomplete sampling of the MAYV population, the possibility that we have underestimated the level of ongoing recombination among circulating strains should also be considered. Despite these concerns, the emergence during the past decade of two MAYV recombinants, herein classified as a new “hybrid” L/D genotype, between genotypes that have historically been circulating in two distant and poorly connected geographic areas, may be indicative of major shifts in the virus ecology and evolutionary dynamic.

Recombination in RNA viruses can be of major evolutionary significance. Recombination processes allows viruses to acquire key adaptive mutations in a single step that might enhance fitness, host tropism, or virulence^29^, such as the potential cross-species transmission effects of the recently identified CHIKV recombinant strain^27^. The ability and opportunity of MAYV to recombine are likely to provide a broader and more rapid search through the viral fitness landscape, thereby facilitating host adaptation^29^. Our *in silico* viral adaptation analyses suggested enhanced human adaptability of the recombinant *E1* gene as compared to non-recombinant genotype D. Such analyses corroborated previous reports of *Ae. aegypti* mosquito as a competent vector for MAYV based on successful *in vitro* replication of MAYV in the *Aedes spp*.^21, 56^. Further *in vitro* investigation will be needed to determine whether recombinant MAYV strains display increased ability to infect this urban-dwelling mosquito and may, therefore, represent a novel arboviral threat to urban populations. On the other hand, our results indicate that, prior to its presence in Haiti, MAYV had been essentially in evolutionary stasis – possibly due to its persistence at a sub-epidemic level in a yet-to-be discovered animal reservoir within the Amazon basin – remaining largely undetected and, consequently, neglected^3, 11, 57^. Arboviruses such as ZIKV, DENV, and CHIKV have exhibited this pattern, circulating for extensive periods in South America, but recently emerging as a concern following their spread to areas such as the Caribbean and USA^12–14^. Over the past few years, Haiti in particular has been a hotspot for the emergence of non-endemic arboviral pathogens^8, 53, 58, 59^. A proactive approach to increase our knowledge on the epidemiology and distribution of occurring and re-occurring arboviruses in this region *will be crucial* to implement countermeasures for novel health threats and their subsequent dissemination in neighboring countries.

## Methods

Methods are briefly outlined below. A detailed description of the software and methods used for the phylogenetic analyses are given in Supplementary Methods. Full-genome sequences of 33 available MAYV strains with specified geographical locations, time of isolation, and source were downloaded from GenBank (Table S1). Alignments of the full-genome sequences, fragments of the genome corresponding to recombinant regions, and each non-structural (*nsP1*, *nsP2*, *nsP3*, *nsP4*) and major structural (*CP*, *E3*, *E2*, *E1*) gene region were visually inspected and optimized prior to analysis. Phylogenetic signal was assessed for each multiple sequence alignment by likelihood mapping analysis and calculating the number of parsimony informative sites (Table S2). We also ruled out substitution saturation by plotting pair-wise transition and transversion nucleotide changes versus genetic distances, which resulted in the expected linear increase for each data set (Figure S6). Phylogenetic-based methods implemented in several different publicly available software packages were used to detect recombination and associated breakpoints. As recombination signal was identified with confidence and was robust, according to a suite of recombination detection tests^31, 34^, additional maximum likelihood based analyses were carried out to determine whether recombination-mediated diversification was driven by selective pressures^60^, such as adaptation to host-specific codon usage. Spatiotemporal dynamics of MAYV gene flow among the various sampled locations within South America and the Caribbean were assessed with the Bayesian phylogeography framework^50^. The presence of sufficient temporal signal (measurable evolution between sampling times), required for molecular clock calibration within this framework, was assessed by linear regression analysis of root-to-tip genetic distance (using trees inferred by maximum likelihood) against sampling time for individual partitioned sequences (genes and recombination-based fragments)^61^. Individual recombinant regions without temporal signal (indicated by a negative slope of the best-fit regression line) were excluded from Bayesian molecular clock analysis (Figure S5). Different demographic priors (constant, Bayesian Skyline plot^62^ and Bayesian Skygrid^49^), molecular clock models (strict vs. relaxed^63^), and data partitions schemes were evaluated within the Bayesian framework by calculating Bayes Factors (Table S3)^64, 65^. Care was taken to determine the impact of sequence sample size on migration inferences by linear regression analysis. Spatiotemporal data and inferences of the timing and location of recombination events were used to inform hypotheses regarding the role of recent human mobility patterns in facilitating recombination and spread of the virus.

## Electronic supplementary material

Supplementary Information
