# Supplementary material for: Emergence of recombinant Mayaro virus strains from the Amazon basin
Source: Sci Rep. 2017 Aug 18;7:8718. doi: 10.1038/s41598-017-07152-5 (PMC5562835; doi:10.1038/s41598-017-07152-5)
Supplement: Supplementary file 1 — Supplementary Information [file 41598_2017_7152_MOESM1_ESM.pdf]

TITLE: Emergence of recombinant *Mayaro virus* strains from the Amazon basin

AUTHORS: Carla Mavian[1,2]*, Brittany D. Rife[1,2]*, James Jarad Dollar[1,2], Eleonora Cella[3], Massimo Ciccozzi[3,4], Mattia C. F. Prosperi[5], John Lednicky[1,6], J. Glenn Morris Jr.[1,7], Ilaria Capua[8]*, Marco Salemi[1,2]*.

AFFILIATIONS

[1]Emerging Pathogens Institute University of Florida, Gainesville FL, USA

[2]Department of Pathology, Immunology and Laboratory Medicine, College of Medicine, University of Florida, Gainesville FL, USA

[3]Department of Infectious, Parasitic and Immune-Mediated Diseases, Istituto Superiore di Sanità, Rome, Italy

[4]Unit of Clinical Pathology and Microbiology, University Campus Biomedico of Rome, Italy

[5]Department of Epidemiology, University of Florida, Gainesville FL, USA

[6] Department of Environmental and Global Health, College of Public Health and Health Professions, University of Florida, Gainesville FL, USA

[7] Department of Medicine, College of Medicine, University of Florida, Gainesville FL, USA

[8]One Health Center of Excellence, University of Florida, Gainesville FL, USA.

*Joint first or senior authors

CORRESPONDING AUTHORS

Correspondence to Marco Salemi: salemi@pathology.ufl.edu and Ilaria Capua: icapua@ufl.edu

KEY WORDS: *Mayaro virus*, alphavirus, recombination, selection, Bayesian phylogeography, phylodynamic, Brazil, Haiti.

FULL MATERIALS AND METHODS

Sequence selection and alignment

The analyses described in the study are based on the full-genome sequences of 33 MAYV isolates obtained from the GenBank database (https://www.ncbi.nlm.nih.gov/genbank/). Full-genome GenBank accession numbers, MAYV isolates names, internal IDs (renamed for convenience), geographical location, source, and year associated with isolation are reported in **Table S1**. Full-genome sequences, fragments of the genome corresponding to recombinant regions, and each major gene region (NSP1, NSP2, NSP3, NSP4, capsid, E3, E2, E1) of MAYV were aligned using the MUSCLE algorithm implemented in MEGA7 (available from http://www.megasoftware.net/) and manually edited to codon-based nucleotide alignments [1-3]. Alignments are available upon request.

Detection of recombination

The presence of conflicting phylogenetic signals (i.e. distinct tree topologies compatible with the same set of aligned sequences) in the MAYV full genome data set was investigated, first, by inferring networks using split decomposition, neighbor-net, consensus network and super networks methods [4,5], implemented in SplitsTree4[4] (available from http://www.splitstree.org/). Significant presence of recombination signal was then assessed with the pairwise homoplasy index (Phi) test[5] in SplitsTree4. Identification of putative recombinant strains, potential parental sequences, and associated breakpoints were performed using the RDP, GENECONV, BootScan, MaxChi, CHIMAERA, SIScan, and 3Seq algorithms implemented in the RDP4[6] software (available from http://web.cbio.uct.ac.za/~darren/rdp.html). Statistical evidence of recombination was indicated by p-values < 0.05. Default settings were used with linear genome specification [6]. Recombination events were considered as such if supported by at least six of the seven algorithms used. Recombination plots were drawn with RDP and bootscanning, implemented in RDP4 and Simplot (available from http://sray.med.som.jhmi.edu/SCRoftware/simplot/)[7]. Two recombination events were detected and RDP4 was also used to infer the genomic location of the recombination breakpoints. The 99% confidence interval (CI) for the first recombinant breakpoint at the 5' region included nucleotide (nt) positions 113 - 129; however, the actual breakpoint was undetermined, likely because the

recombination event was shared by two strains. We, therefore, approximated the breakpoint at position 97, the mean of the CI. The same indeterminacy was observed for the beginning of the second recombinant fragment at the 3' region, with 99% CI including nts 10,788 - 10,912, the mean being position 10,850.

Phylogenetic signal and ML phylogeny inference

Evaluation of the presence of phylogenetic signal satisfying resolved phylogenetic relationships among MAYV isolated sequences was performed for full genomes, individual genes, and recombinant regions using IQ-TREE (available from http://www.iqtree.org/), allowing the software to search for all possible quartets using the best-fitting nucleotide substitution model (**Table S2**) [8]. Substitution saturation, which decreases the phylogenetic information contained in the sequences, was assessed using DAMBE6 (available from http://dambe.bio.uottawa.ca/DAMBE/)[9] (**Figure S7**). ML tree reconstruction was performed in IQ-TREE based on the best-fit model chosen according to Bayesian Information Criterion (BIC) [10,11].

Even though the concatenation of sequences from multiple genes is often used in the estimation of the "species" tree, this approach can lead to poor inference of past population dynamics [12] due to varying selective forces on the different regions within the genome. The incorporation of multiple loci in the coalescent framework has been shown to yield more precise and less biased estimates of past population dynamics, especially during time periods for which single locus data are not very informative [13], Therefore, we allowed for varying evolution of each gene independently according to potentially unique patterns of substitution and distinct evolutionary rates. In order to do so, the full genome and non-recombinant regions were partitioned into 11 regions corresponding to each gene and non-coding sequence (non-coding nt region 1= 1-36; NSP1= 37-1,644; NSP2 =1,645-4,038; NSP3 = 4,039-5,511; NSP4 = 5,512-7,365; non-coding region 2 = 7,366-7,424; CP = 7,366-8,198; E3 = 8,199-8,396; E2 = 8,397-9,665; 6k = 9,666-9,845; E1 = 9,846-11,153). Statistical robustness for internal branching order in the phylogeny was assessed by local- and single-branch standard, non-parametric bootstrapping (BS) (1,000 replicates), and Shimodaira–Hasegawa-like approximate likelihood ratio test (SH-aLRT) (1,000 replicates) [10,14,15]. Strong statistical support along the branches was defined as local and/or standard non-parametric BS>75% or SH-

aLRT>0.99, whereas very strong statistical support was defined as local or/and standard non-parametric BS>75% and SH-aLRT>0.99.

Selection analysis

Analyses were performed on the five genomic regions individually: nucleotides (nts) 1-96 and 10850-11151, corresponding to the recombinant regions obtained from the first recombination event; nts 133-929, corresponding to the second recombination event; and the two non-recombinant regions (nts 97-132 and 930-10,849). Full-length genes were also analyzed, with the exception of E1 gene, for which the 5' nucleotide region overlaps with the 5' region of alternatively spliced TransFrame protein-coding gene, located at position 9,808-9,890. This region was removed from the gene-specific alignment [16].

The HyPhy [17] algorithms implemented in the Adaptive Evolution Server (http://www.datamonkey.org/) were used to estimate the ratio (ω) of non-synonymous (dN) to synonymous (dS) substitution rates for each codon, with $\omega<0$ indicating purifying, or negative, selection and $\omega >0$ indicating diversifying, or positive, selection [18]. The following selection analyses were conducted according to the best-fit substitution model, also determined within the Server: adaptive branch-site random effects likelihood (aBSREL)[19] model for the detection of lineage-specific selection, fast unconstrained Bayesian approximation (FUBAR)[20] for inferring site-specific pervasive selection, Bayesian unrestricted test for episodic diversifying selection (BUSTED)[21] across the region of interest, and the mixed effects model of evolution (MEME) to identify episodic selection at individual sites [22]. Sites were considered to have experienced statistically significant positive or negative selection based on the following cut-offs: LRT $p \leq 0.05$ for BUSTED and aBSREL and posterior probability (PP) > 0.90 for FUBAR, and LRT ≤ 0.05 for MEME. Because inference of diversifying selection can be misled by the incorporation of recombinant sequences [23,24], the recombinant segments for each of these two genes were analyzed separately for each selection model, with the exception of MEME, which has shown to be robust to the presence of recombination [22] . Co-evolving codon sites in the recombinant proteins NSP1 and E1 were identified using the Bayesian graphical model ("Spidermonkey"), also implemented in the Adaptive Evolution Server, which identifies conditional co-evolutionary

dependencies from reconstructed substitutions at each branch/site combination [25]. Pairs of interactions with PP>0.5 were considered co-evolving with statistical significance.

Codon usage analysis

The codon adaption index (CAI) was determined in order to evaluate the relative adaptability of the codon usage of a gene towards that of highly expressed genes within a given host [26]. The CAI values of MAYV genomes were analyzed in the context of the following: *Homo sapiens* as host, *Ixodes Pararicinus* and *Saimiri sciureus* as potential natural reservoirs; and *Aedes aegypti, Aedes albopictus, Culex pipiens pipiens*, and *Culex pipiens quinquefasciatus* as potential urban vectors. The codon usage of MAYV in the context of its principal source of isolation, *Haemagogus janthinomys*, and specific to the *Ixodes spp.* of tick belonging to the northern region of Brazil *(I. paránaensis, loricatus, boliviensis, and affinis)* was not available; therefore, we based our analysis on the codon usage of *I. pararicinus,* known as the "South American cattle tick", distributed in the temperate and sub-tropical regions of southern Brazil, Uruguay, and Argentina[27,28].The codon usage tables were downloaded individually for each organism tested from the codon usage database (http://www.kazusa.or.jp/codon/) in NCBI-GenBank Flat File format (Release 160.0 June 15 2007) and manually edited in order to be added to the local database in DAMBE6, allowing calculation of the CAI values within DAMBE6 [9].

Bayesian coalescent inference

The presence of temporal signal in each data set was assessed using TempEst v1.5 (available from http://tree.bio.ed.ac.uk/software/tempest/) [29] (**Figure S5**). Tree reconstruction, using molecular clock dating, of MAYV full-genome and genomic fragments corresponding to the non-recombinant regions (97-132 nt plus 930-10,849 nt) was performed with the Bayesian coalescent framework implemented in the BEAST v1.8.3 software package (http://beast.bio.ed.ac.uk/) [30,31]. Depending on the genomic region analyzed, Markov chain Monte Carlo (MCMC) samplers were run for 100, 200 or 500 million generations to achieve proper mixing of the Markov chain. Proper mixing of the MCMC was evaluated by calculating the Effective Sampling Size (ESS) of the parameter estimates with TRACER v1.6 in the BEAST package. ESS >200 (after 10% a burn-in) were considered robust. The HKY substitution model [32] was used with

empirical base frequencies and gamma distribution of site-specific rate heterogeneity. The constant size demographic model was tested against the Bayesian Skygrid (BSG) [13] or Bayesian Skyline Plot (BSP) [33], the latter two models corresponding to partitioned (BSG) or non-partitioned sequence alignments (BSP), in order to rule out spurious changes in effective population size inferred by a non-parametric model that would in turn impact timing of divergence events [34]. Additionally, for each demographic model, we assessed the fit of the strict and relaxed, uncorrelated (lognormal distribution among branches) molecular clock models. The choice of dataset partitioning was dictated by differences observed in tree topology during ML reconstruction of the different genes and because the partitioning method allows each gene and non-coding sequence to evolve according to its own substitution model and evolutionary rate, improving the estimation of past population dynamics, while still maintaining the underlying phylogenetic tree topology by linking the trees [13]. Marginal likelihood estimates (MLE) for Bayesian model testing were obtained using path sampling (PS) and stepping-stone sampling (SS) methods [31,35]. The strength of evidence against the null hypothesis (H_0) was evaluated via MLE comparison with the more complex model (H_A), referred to as the Bayes Factor (BF), wherein *ln*BF<2 indicates no evidence against H_0; 2–6 - weak evidence; 6–10 - strong evidence, and >10 indicates very strong evidence [36]. When comparing nested models, the best-fitting model chosen for Bayesian phylogenetic and phylogeographic inferences (see next section) was the BSG demographic model with a strict molecular clock (**Table S3**). The posterior sampled trees were summarized within the maximum clade credibility (MCC) tree, which was identified using TreeAnnotator v1.8.3 in the BEAST package, specifying a burn-in of 10% and median node heights [31]. Trees were edited graphically in FigTree v1.4.2 (available from http://tree.bio.ed.ac.uk/software/figtree/) and DensiTree v2 (available from https://www.cs.auckland.ac.nz/~remco/DensiTree/), and later in PowerPoint (Microsoft) for publication purposes. Nodes with PP ≥ 0.99 were considered to be evidence of statistically significant phylogenetic relationships [31,37]. Xml files are available upon request.

Bayesian phylogeography analysis

Bayesian phylogeography analysis was performed with BEAST [30] using a discrete trait, asymmetric transition (migration) model with the strict molecular clock, non-parametric demographic (BSG), and Bayesian stochastic search variable selection (BSSVS) models. Discrete MCC tree data were merged with

user-specified longitude and latitude data for each sampling location for generation of the kml file required for graphical representation of migration patterns were performed with SPREAD (www.kuleuven.be/aidslab/phylogeography/SPREAD.html) software [38], and Google Maps (https://mapstyle.withgoogle.com). BF values of 3-20 indicate positive evidence, but BF of 20–150 and >150 indicate strong and very strong evidence, respectively [38,39]. In order to reduce false positives migration correlations between MAYV isolates, as these may impact future prevention strategies, only migration rates with BF>10 according to the BSSVS, were used for final graphical interpretation. As a uniform sampling scheme for sequencing was difficult and proportionality of sequence number to prevalence unknown or uncertain for different locations, linear regression analysis was used to determine the influence of potential sampling bias on migration rate estimates. Log_2 transformation of migration rates between locations (asymmetric) and donor and recipient location sequence sample sizes were used to determine the R^2 value of association (**Figure S6**). An accessibility map indicating the estimated travel time (using land road/off road or water navigable river, lake and ocean) in minutes, hours and days to the nearest city of 50,000 or more people (year 2000), was obtained from the ArcGIS database (https://www.arcgis.com) and based on a previously published and available dataset (https://tiles.arcgis.com/tiles/P8Cok4qAP1sTVE59/arcgis/rest/services/Accessibility_Travel_time_to_Major_Cities/MapServer) [40]. Additional graphical representation of Haitian earthquake refugee flux to Brazil (and Peru) and Brazilian personnel contribution to the United Nations Stabilization Mission In Haiti (MINUSTAH) following the 2010 earthquake in Haiti were recreated manually based on previously published data [41-43].

SUPPLEMENTARY REFERENCES

1 Kumar, S., Stecher, G. & Tamura, K. MEGA7: Molecular Evolutionary Genetics Analysis Version 7.0 for Bigger Datasets. *Mol Biol Evol* **33**, 1870-1874, doi:10.1093/molbev/msw054 (2016).

2 Edgar, R. C. MUSCLE: multiple sequence alignment with high accuracy and high throughput. *Nucleic Acids Res* **32**, 1792-1797, doi:10.1093/nar/gkh340 (2004).

3 Edgar, R. C. MUSCLE: a multiple sequence alignment method with reduced time and space complexity. *BMC Bioinformatics* **5**, 113, doi:10.1186/1471-2105-5-113 (2004).

4 Huson, D. H. & Bryant, D. Application of phylogenetic networks in evolutionary studies. *Mol Biol Evol* **23**, 254-267, doi:10.1093/molbev/msj030 (2006).

5 Bruen, T. C., Philippe, H. & Bryant, D. A simple and robust statistical test for detecting the presence of recombination. *Genetics* **172**, 2665-2681, doi:10.1534/genetics.105.048975 (2006).

6 Martin, D. P., Murrell, B., Golden, M., Khoosal, A. & Muhire, B. RDP4: Detection and analysis of recombination patterns in virus genomes. *Virus Evol* **1**, vev003, doi:10.1093/ve/vev003 (2015).

7 Lole, K. S. *et al.* Full-length human immunodeficiency virus type 1 genomes from subtype C-infected seroconverters in India, with evidence of intersubtype recombination. *J Virol* **73**, 152-160 (1999).

8 Schmidt, H. A., Strimmer, K., Vingron, M. & von Haeseler, A. TREE-PUZZLE: maximum likelihood phylogenetic analysis using quartets and parallel computing. *Bioinformatics* **18**, 502-504 (2002).

9 Xia, X. & Xie, Z. DAMBE: software package for data analysis in molecular biology and evolution. *J Hered* **92**, 371-373 (2001).

10 Nguyen, L. T., Schmidt, H. A., von Haeseler, A. & Minh, B. Q. IQ-TREE: a fast and effective stochastic algorithm for estimating maximum-likelihood phylogenies. *Mol Biol Evol* **32**, 268-274, doi:10.1093/molbev/msu300 (2015).

11 Trifinopoulos, J., Nguyen, L. T., von Haeseler, A. & Minh, B. Q. W-IQ-TREE: a fast online phylogenetic tool for maximum likelihood analysis. *Nucleic Acids Res* **44**, W232-235, doi:10.1093/nar/gkw256 (2016).

12 Kubatko, L. S. & Degnan, J. H. Inconsistency of phylogenetic estimates from concatenated data under coalescence. *Syst Biol* **56**, 17-24, doi:10.1080/10635150601146041 (2007).

13 Gill, M. S. *et al.* Improving Bayesian population dynamics inference: a coalescent-based model for multiple loci. *Mol Biol Evol* **30**, 713-724, doi:10.1093/molbev/mss265 (2013).

14 Guindon, S. *et al.* New algorithms and methods to estimate maximum-likelihood phylogenies: assessing the performance of PhyML 3.0. *Syst Biol* **59**, 307-321, doi:10.1093/sysbio/syq010 (2010).

15 Shimodaira, H. & Hasegawa, M. Multiple comparisons of log-likelihoods with applications to phylogenetic inference. *Molecular Biology and Evolution* **16**, 1114-1116 (1999).

16 Firth, A. E., Chung, B. Y., Fleeton, M. N. & Atkins, J. F. Discovery of frameshifting in Alphavirus 6K resolves a 20-year enigma. *Virol J* **5**, 108, doi:10.1186/1743-422X-5-108 (2008).

17 Pond, S. L., Frost, S. D. & Muse, S. V. HyPhy: hypothesis testing using phylogenies. *Bioinformatics* **21**, 676-679, doi:10.1093/bioinformatics/bti079 (2005).

18 Pond, S. L. & Frost, S. D. Datamonkey: rapid detection of selective pressure on individual sites of codon alignments. *Bioinformatics* **21**, 2531-2533, doi:10.1093/bioinformatics/bti320 (2005).

19 Kosakovsky Pond, S. L. *et al.* A random effects branch-site model for detecting episodic diversifying selection. *Mol Biol Evol* **28**, 3033-3043, doi:10.1093/molbev/msr125 (2011).

20 Murrell, B. *et al.* FUBAR: a fast, unconstrained bayesian approximation for inferring selection. *Mol Biol Evol* **30**, 1196-1205, doi:10.1093/molbev/mst030 (2013).

21 Murrell, B. *et al.* Gene-wide identification of episodic selection. *Mol Biol Evol* **32**, 1365-1371, doi:10.1093/molbev/msv035 (2015).

22 Murrell, B. *et al.* Detecting individual sites subject to episodic diversifying selection. *PLoS Genet* **8**, e1002764, doi:10.1371/journal.pgen.1002764 (2012).

23 Anisimova, M., Nielsen, R. & Yang, Z. Effect of recombination on the accuracy of the likelihood method for detecting positive selection at amino acid sites. *Genetics* **164**, 1229-1236 (2003).

24 Shriner, D., Nickle, D. C., Jensen, M. A. & Mullins, J. I. Potential impact of recombination on sitewise approaches for detecting positive natural selection. *Genet Res* **81**, 115-121 (2003).

25 Poon, A. F., Lewis, F. I., Pond, S. L. & Frost, S. D. An evolutionary-network model reveals stratified interactions in the V3 loop of the HIV-1 envelope. *PLoS Comput Biol* **3**, e231, doi:10.1371/journal.pcbi.0030231 (2007).

26 Sharp, P. M. & Li, W. H. The codon Adaptation Index--a measure of directional synonymous codon usage bias, and its potential applications. *Nucleic Acids Res* **15**, 1281-1295 (1987).

27 Keirans, J. E., Clifford, C. M., Guglielmone, A. A. & Mangold, A. J. Ixodes (Ixodes) pararicinus, n. sp. (Acari: Ixodoidea: Ixodidae), a South American cattle tick long confused with Ixodes ricinus. *J Med Entomol* **22**, 401-407 (1985).

28 Evans, D. E., Martins, J. R. & Guglielmone, A. A. A review of the ticks (Acari, ixodida) of Brazil, their hosts and geographic distribution - 1. The state of Rio Grande do Sul, southern Brazil. *Mem Inst Oswaldo Cruz* **95**, 453-470 (2000).

29 Rambaut, A., Lam, T. T., Max Carvalho, L. & Pybus, O. G. Exploring the temporal structure of heterochronous sequences using TempEst (formerly Path-O-Gen). *Virus Evol* **2**, vew007, doi:10.1093/ve/vew007 (2016).

30 Drummond, A. J., Suchard, M. A., Xie, D. & Rambaut, A. Bayesian phylogenetics with BEAUti and the BEAST 1.7. *Mol Biol Evol* **29**, 1969-1973, doi:10.1093/molbev/mss075 (2012).

31 Drummond, A. J. & Rambaut, A. BEAST: Bayesian evolutionary analysis by sampling trees. *BMC Evol Biol* **7**, 214, doi:10.1186/1471-2148-7-214 (2007).

32 Hasegawa, M., Kishino, H. & Yano, T. Dating of the human-ape splitting by a molecular clock of mitochondrial DNA. *J Mol Evol* **22**, 160-174 (1985).

33 Strimmer, K. & Pybus, O. G. Exploring the demographic history of DNA sequences using the generalized skyline plot. *Mol Biol Evol* **18**, 2298-2305 (2001).

34 Matthew D. Hall, M. E. J. W., Andrew Rambaut. The effects of sampling strategy on the quality of reconstruction of viral population dynamics using Bayesian skyline family coalescent methods. *Virus Evol* **2** (2016).

35 Baele, G. *et al.* Improving the Accuracy of Demographic and Molecular Clock Model Comparison While Accommodating Phylogenetic Uncertainty. *Molecular Biology and Evolution* **29**, 2157-2167, doi:10.1093/molbev/mss084 (2012).

36 Xie, W., Lewis, P. O., Fan, Y., Kuo, L. & Chen, M. H. Improving marginal likelihood estimation for Bayesian phylogenetic model selection. *Syst Biol* **60**, 150-160, doi:10.1093/sysbio/syq085 (2011).

37 Remco R. Bouckaert, J. H. DensiTree 2: Seeing Trees Through the Forest. *biorxiv* (2014).

38 Bielejec, F., Rambaut, A., Suchard, M. A. & Lemey, P. SPREAD: spatial phylogenetic reconstruction of evolutionary dynamics. *Bioinformatics* **27**, 2910-2912, doi:10.1093/bioinformatics/btr481 (2011).

39 Robert E. Kass, A. E. R. Bayes Factors. *Journal of the American Statistical Association* **90**, 773-795 (1995).

40 Nelson, A. Estimated travel time to the nearest city of 50,000 or more people in year 2000. *Global Environment Monitoring Unit - Joint Research Centre of the European Commission, Ispra, Italy* http://bioval.jrc.ec.europa.eu/products/gam (2008).

41 Rawlinson, T. *et al.* From Haiti to the Amazon: public health issues related to the recent immigration of Haitians to Brazil. *PLoS Negl Trop Dis* **8**, e2685, doi:10.1371/journal.pntd.0002685 (2014).

42 Nations, U. Troop and police contributors archive (1990 - 2014) http://www.un.org/en/peacekeeping/resources/statistics/contributors_archive.shtml.

43 Nations, U. Restoring a secure and stable environment. http://www.un.org/en/peacekeeping/missions/minustah/index.shtml.

SUPPLEMENTARY TABLES

Table S1. MAYV isolate information.

GenBank accession number	GenBank ID	Internal ID	Geographical location	Source	Year
KX496990	Haiti-1/2015	HAITI15	Haiti	*Homo sapiens*	2015
KT754168	BeAr20290	1BR60	Pará, Brazil	*Haemagogus spp.*	1960
KT818520	BR/SJRP/LPV01/2015	2BR14	São Paulo Brazil	*Homo sapiens*	2014
KP842820	BeAr30853	3BR61	Pará, Brazil	*Ixodes spp.*	1961
KP842819	BeH256	4BR55	Pará, Brazil	*Homo sapiens*	1955
KP842818	BeAr505411	5BR91	Pará, Brazil	*Haemagogus janthinomys*	1991
KP842817	FVB0069	6BO06	Bolivia	*Homo sapiens*	2006
KP842816	FPI0179	7PE11	Iquitos, Peru	*Homo sapiens*	2011
KP842815	FPI1761	8PE11	Iquitos, Peru	*Homo sapiens*	2011
KP842814	FVB0112	9BO06	Bolivia	*Homo sapiens*	2006
KP842813	FPY0046	10PE11	Yurimaguas, Peru	*Homo sapiens*	2011
KP842812	FMD3213	11PE10	Puerto Maldonado, Peru	*Homo sapiens*	2010
KP842811	FMD0641	12PE05	Puerto Maldonado, Peru	*Homo sapiens*	2005
KP842810	TRVL15537	13TT57	Trinidad and Tobago	*Haemagogus janthinomys*	1957
KP842809	BeH186258	14BR70	Brazil	*Homo sapiens*	1970
KP842808	IQU3056	15PE00	Loreto, Peru	*Homo sapiens*	2000
KP842807	Ohio	16PE95	Loreto, Peru	*Homo sapiens*	1995
KP842806	FSB1131	17BO06	Bolivia	*Homo sapiens*	2006
KP842805	FSB0319	18BO02	Bolivia	*Homo sapiens*	2002
KP842804	BeAn337622	19BR78	Pará, Brazil	Non-human primate	1978
KP842803	BeH343148	20BR78	Pará, Brazil	*Homo sapiens*	1978
KP842802	BeAn343102	21BR78	Pará, Brazil	Non-human primate	1978
KP842801	IQE2777	22PE06	Loreto, Peru	*Homo sapiens*	2006
KP842800	ARV0565	23PE95	San Martin, Peru	*Homo sapiens*	1995
KP842799	MAYV15A	24VE10	La Estación Portuguesa, Venezuela	*Homo sapiens*	2010
KP842798	MAYV14A	25VE10	La Estación Portuguesa, Venezuela	*Homo sapiens*	2010
KP842797	MAYV13A	26VE10	La Estación Portuguesa, Venezuela	*Homo sapiens*	2010
KP842796	MAYV12A	27VE10	La Estación Portuguesa, Venezuela	*Homo sapiens*	2010
KP842795	MAYV11A	28VE10	La Estación Portuguesa, Venezuela	*Homo sapiens*	2010
KP842794	MAYV16A	29VE10	La Estación Portuguesa, Venezuela	*Homo sapiens*	2010
KM400591	Acre27	30BR04	Acre, Brazil	*Homo sapiens*	2004
KJ013266	BNI-1	31GF13	French Guiana	*Homo sapiens*	2013
DQ001069	MAYLC	35GF99	French Guiana	*Homo sapiens*	1999

Table S2. Mayaro virus genome-wide phylogenetic resolution analysis and best-fit model chosen according to Bayesian Information (BIC)

Gene or region	Constant sites (%)	Parsimony informative sites (%)	Phylogenetic noise (%)	Evolutionary model
Full genome	77.7	16.2	3.3	GTR+G
NSP1	81.5	13.6	12.5	TN93+G
NSP2	77.2	16.6	5.0	TN93+G
NSP3	74.9	19.6	10.0	K2+G
NSP4	78.0	16.6	14.8	K2+G
CP	80.9	14.9	20.8	TN93+G
E3	77.8	18.2	22.6	K2+G
E2	76.9	1.7	6.2	K2+G
E1	73.6	19.6	18.1	K2+G
D1+D2[a]	80.5	11.3	51.7	K2P+G4
D3[b]	84.6	11.0	25.2	TIM2e+G4
L1s+L1l[c]	77.1	16.8	2.9	GTR+G4

[a]concatenated genomic fragments resulting from the first recombination event . [b]genomic fragment resulting from the second recombination event. [c]concatenated non-recombinant fragments of the genome

Table S3. Bayes factor (BF) comparison of nested molecular clock and Bayesian demographic models. The natural logarithm of the BF was used for comparison of the strict (SC) and uncorrelated relaxed lognormal (UCLN) molecular clock models and the constant and non-parametric Bayesian skyline plot (BSP) and skygrid (BSG) demographic models within BEAST 1.8.3.

	SC Constant	UCLN Constant	SC BSP	UCLN BSP	SC BSG	UCLN BSG
SC Constant		-261.254	4.482	-	79.276	-
UCLN Constant	-262.142		-	5.692	-	55.111
SC BSP	4.074	-		-260.044	74.794	-
UCLN BSP	-	6.260	-259.956		-	59.106
SC BSG	78.915	-	74.841	-		-279.727
UCLN BSG	-	65.366	-	59.106	-275.692	

Path sampling (upper triangle)

Stepping stone sampling (lower triangle)

Table S4. Bayesian TMRCAs estimates according to different demographic models. The TMRCA best fitting the data is given in bold.

Clock Model	Coalescent Prior	TMRCAs[a]		
		Node A	Node B	Node C
Strict	Const	2007.8 (2002.2-2012.1)	1947.4 (1939-1953.8)	1948.9 (1924.7-1967.2)
Strict	Skyline	2008.7 (2004.3-2012.5)	1947.5 (1938.7-1953.7)	1948.3 (1922.4-1967.4)
**Strict**	**Skygrid**	**2007.6 (2001.7-2012.2)**	**1946.8 (1937-1953.6)**	**1945.9 (1919-1967.1)**

[a]Node A corresponds to common ancestor node of recombinant sequences, Node B to ancestor node of 3BR61, and Bode C to ancestor node of 30BR04.

Table S5. Bayes Factor (BF) values and directionality of inferred MAYV migration rates

BF[a]	Country of origin	Country of destination
235	Bolivia	Puerto Maldonado, Peru
46	French Guiana	Pará, Brazil
46	Loreto, Peru	Yurimaguas, Peru
43	Loreto, Peru	La Estación Portuguesa, Venezuela
31	Loreto, Peru	Iquitos, Peru
29	Loreto, Peru	San Martin, Peru
17	French Guiana	Loreto, Peru
16	Loreto, Peru	Bolivia
15	São Paulo, Brazil	Haiti
15	Bolivia	Acre, Brazil
14	Pará, Brazil	São Paulo, Brazil
13	Haiti	São Paulo, Brazil
12	Pará, Brazil	Haiti
9	Trinidad and Tobago	Brazil
6	Brazil	Trinidad and Tobago
6	Loreto, Peru	Acre, Brazil
6	Trinidad and Tobago	French Guiana
5	Brazil	French Guiana
5	Acre, Brazil	Bolivia
5	Puerto Maldonado, Peru	Trinidad and Tobago
4	Puerto Maldonado, Peru	Brazil
4	Puerto Maldonado, Peru	French Guiana
3	Loreto, Peru	French Guiana
3	French Guiana	Trinidad and Tobago

[a]BF ranging from 3-20 was indicative of evidence of a non-zero transition (migration) rate between locations, 20-150 indicative of strong evidence, and >150 indicative of very strong evidence.

SUPPLEMENTARY FIGURES

Figure S1

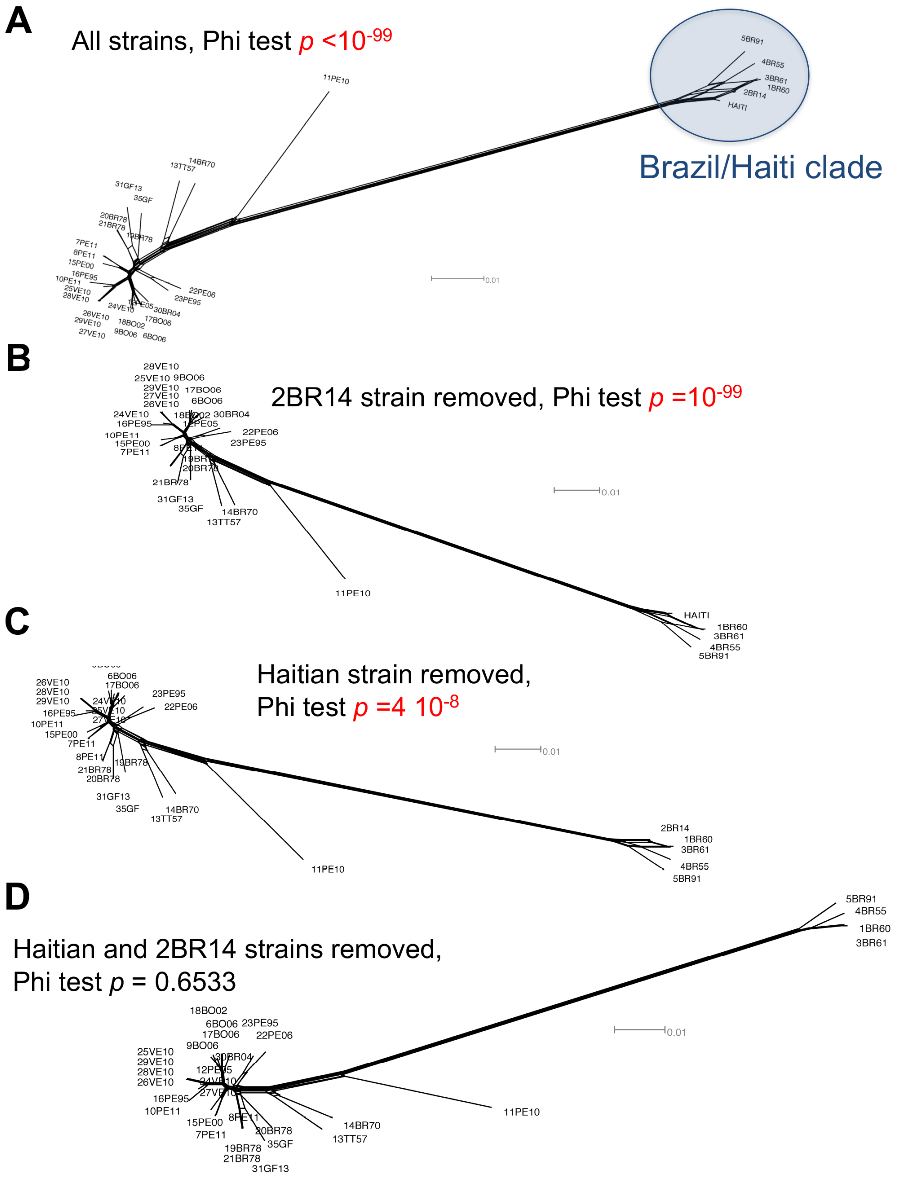

Figure S1. Network graphical modeling test to assess recombination. Network graphs were generated in SplitsTree based on (**A**) all MAYV strains which full genome sequence is available, (**B**) dataset without 2RB14 strain, (**C**) dataset without HAITI15 strain, and (**D**) dataset without 2RB14 and HAITI15 strains. The p values of Phi test of recombination for each combination of dataset are reported in each panel ($p < 0.05$ indicate significance evidence of recombination).

Figure S2

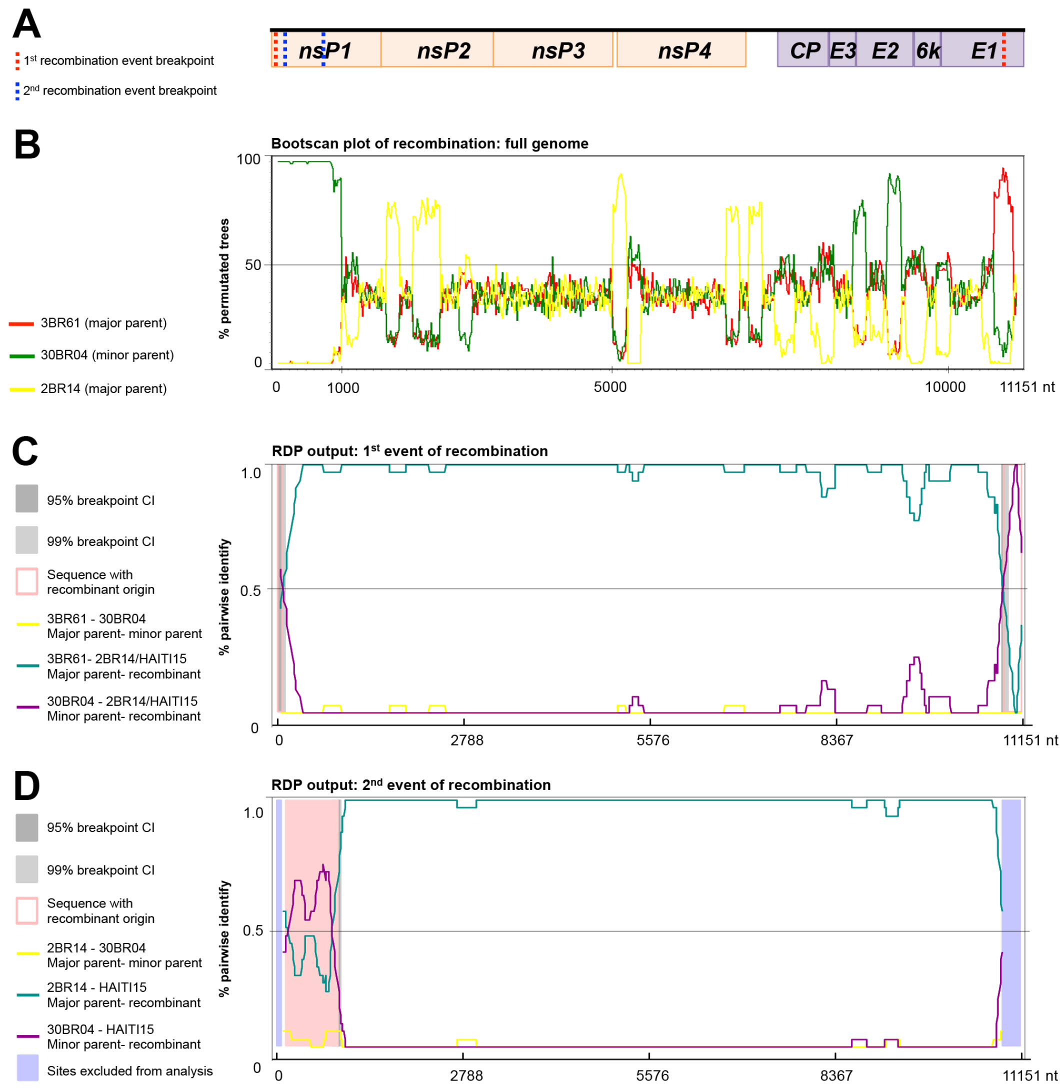

Figure S2. Characterization of the recombination events in MAYV genome. (**A**) Schematic representation of MAYV genome (blue), non-structural genes (blue) and structural genes (pink). Dotted blue and red lines indicate the breakpoints. (**B**) Bootscan recombination plot of the genome of HAITI15 against the genomes of 2BR14 (major parental sequence) in yellow, 3BR61 (major parental sequence of 2BR14) in green and 30BR14 (minor parental sequence) in red representing the recombinant regions. RDP recombination plot for the (**C**) first recombination event and for the (**D**) second recombination event.

Figure S3

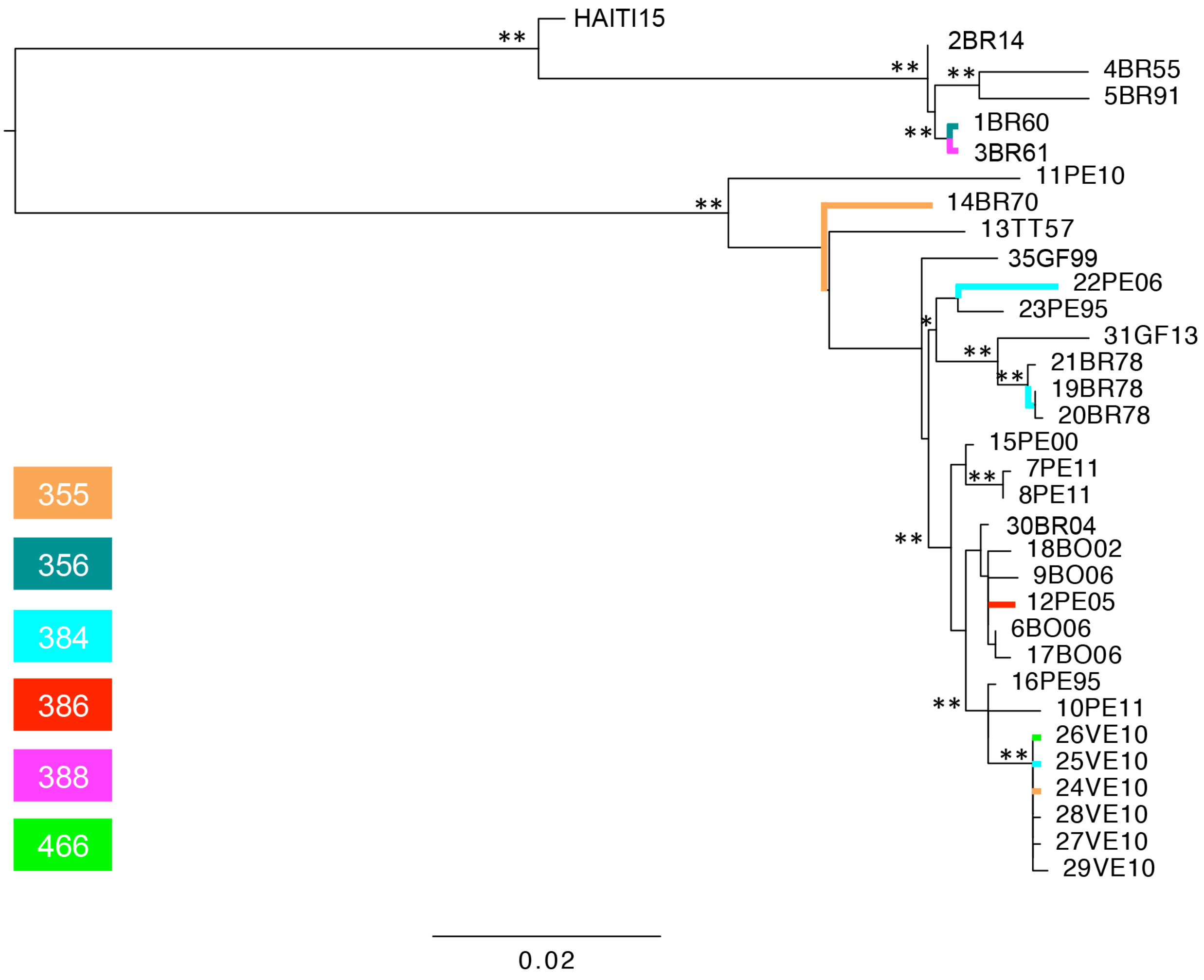

Figure S3. MAYV *nsP1* genealogy depicting amino acid changes for sites under episodic diversifying selection. Six *nsP1* sites were identified using MEME as experiencing episodic diversifying selection (EDS). Branches corresponding to amino acid changes in these sites were determined using parsimonious ancestral state reconstruction using Mesquite and mapped onto the *nsP1* maximum likelihood tree. Amino acid position is given as colored boxes in the left of the figure. The color of each residue box corresponds with the color of the branch experiencing EDS.

Figure S4

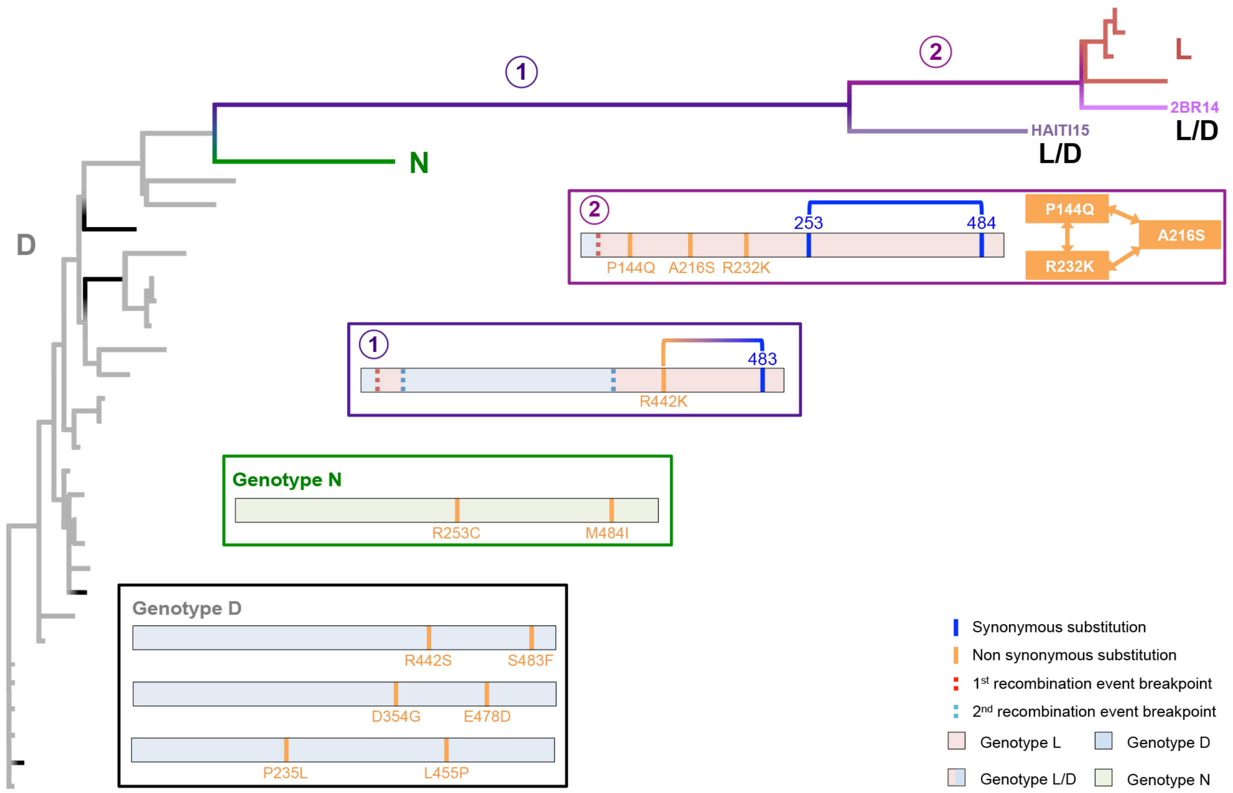

Figure S4. Visualization of co-evolving sites in the recombinant *nsP1* gene. Co-evolution between sites in the recombinant *nsP1* gene was inferred from substitutions occurring along single branches within the fixed maximum likelihood ML tree topology using the Bayesian graphical model implemented in the datamonkey webserver (http://datamonkey.org/). Pairs of co-evolving sites were mapped to branches in the tree and in the schematic representation of the gene for each genotype. Synonymous substitutions are given in blue, non-synonymous substitutions in orange. Branches are colored as follows: branches leading to genotype D in gray, specifically branches with co-evolving sites within genotype D are highlighted in black; branches leading to genotype N in green; branches leading to genotypes L/D and L in dark violet ('recombinant branch 1'); branches leading to HAITI15 strain in light violet; branches leading to genotype L/D 2BR14 and L strain in purple ('recombinant branch 2'); branches leading to 2BR14 strain in light purple; branches leading to genotype L in maroon.

Figure S5

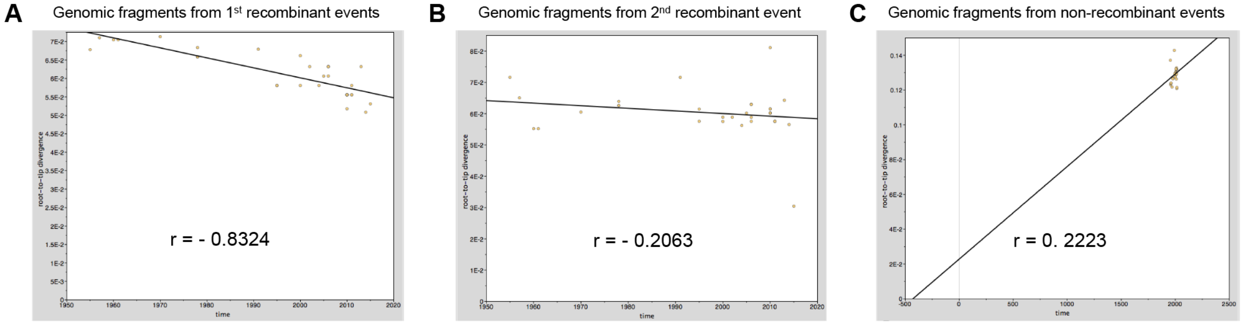

Figure S5. Regression analysis of temporal resolution of MAYV sequence datasets. The plots represent linear regression of root-to-tip genetic distance within the ML phylogeny against sampling time for each taxa. Temporal resolution was assessed using the slope of the regression, with positive slope indicating sufficient temporal signal for (**A**) non-recombinant fragments, and recombinant fragments from the (**B**) first and (**C**) second recombination events. Correlation coefficient “r” are reported for each genomic fragment.

Figure S6

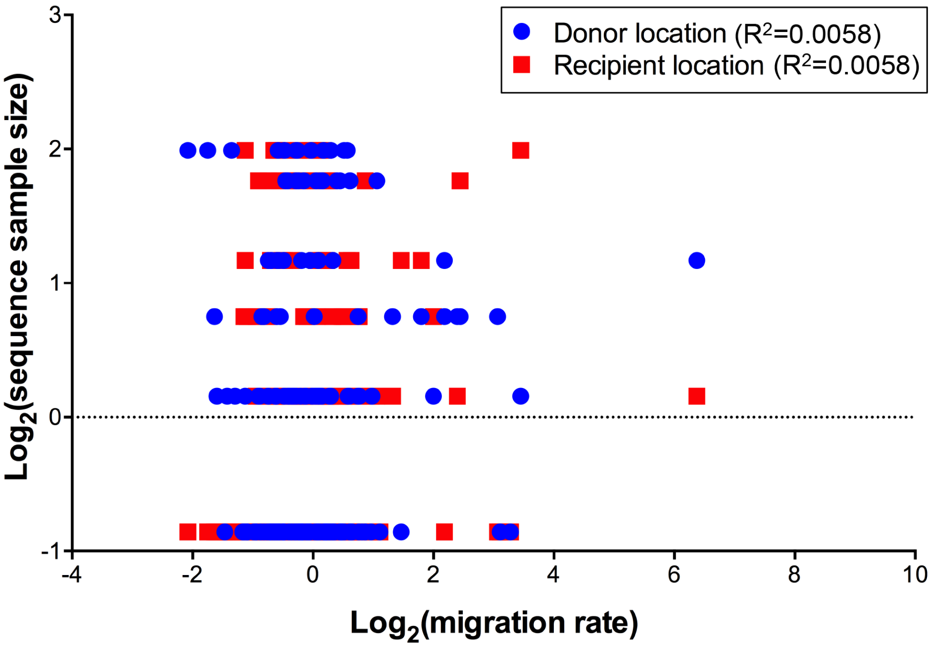

Figure S6. Impact of sequence sample size on inferred migration rates. Linear regression analysis showing correlation between MAYV sequence sample size (strains by location) and location migration rates (BSSV values). Donor locations are showed in blue, while locations recipient in red.

Figure S7

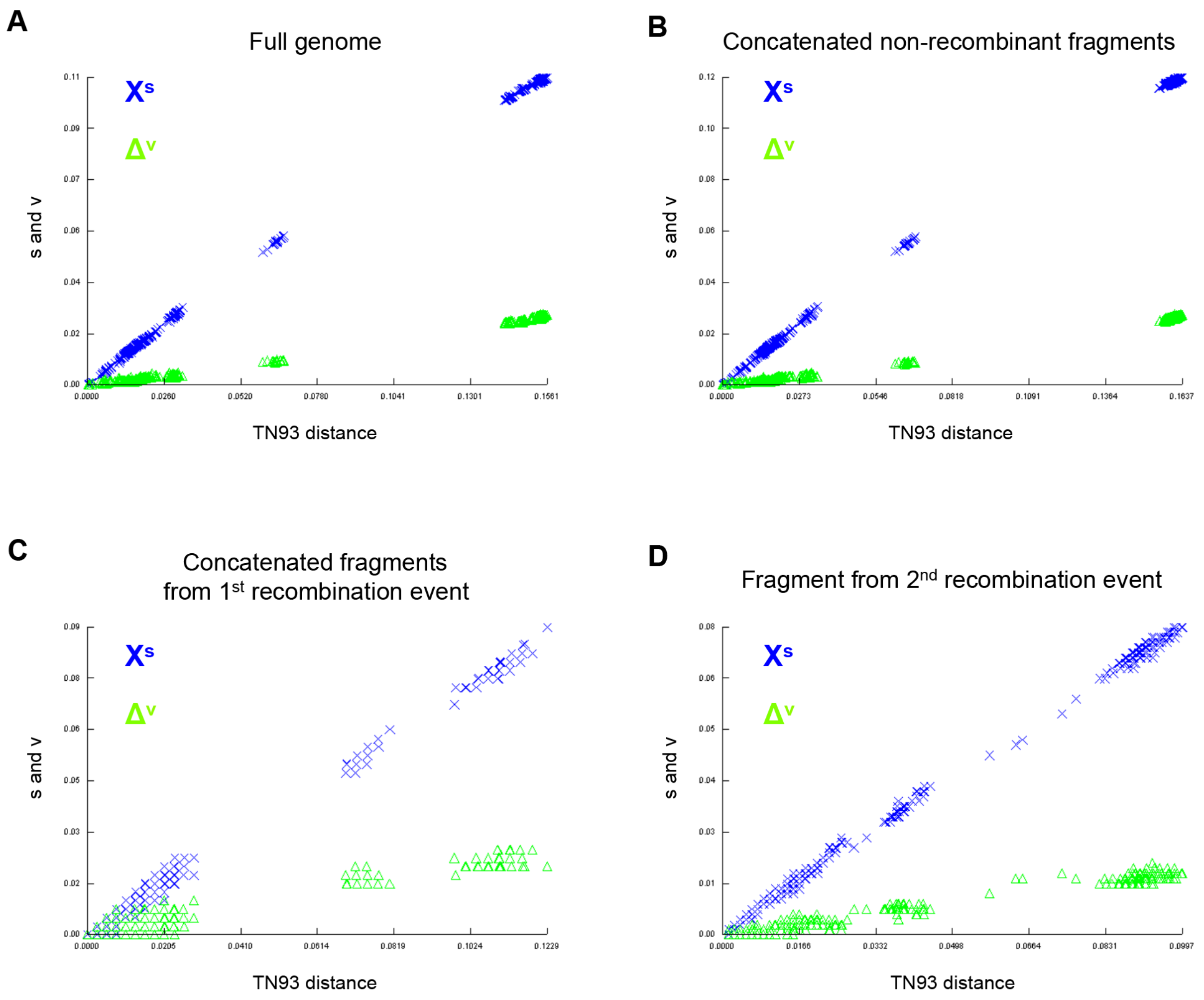

Figure S7. Substitution saturation in MAYV sequence datasets. Scatter plots of pairwise nucleotide transition (s) and transversion (v) substitutions against the Tamura and Nei 1993 (TN93) genetic distance were generated within DAMBE v5 for (**A**) full-genome, (**B**) non-recombinant fragments, and recombinant fragments from the (**C**) first and (**D**) second recombination events.
